# Vitamin D Impacts the Expression of Runx2 Target Genes and Modulates Inflammation, Oxidative Stress and Membrane Vesicle Biogenesis Gene Networks in 143B Osteosarcoma Cells

**DOI:** 10.3390/ijms18030642

**Published:** 2017-03-16

**Authors:** Rama Garimella, Priyanka Tadikonda, Ossama Tawfik, Sumedha Gunewardena, Peter Rowe, Peter Van Veldhuizen

**Affiliations:** 1 Division of Medical Clinical Oncology, The University of Kansas Medical Center, Kansas City, KS 66160, USA; Peter.Vanveldhuizen@va.gov; 2 Departments of Internal Medicine, The University of Kansas Medical Center, Kansas City, KS 66160, USA; prowe@kumc.edu; 3 Orthopedic Surgery, The University of Kansas Medical Center, Kansas City, KS 66160, USA; 4 Dietetics and Nutrition, The University of Kansas Medical Center, Kansas City, KS 66160, USA; surya.priyanka@gmail.com; 5 Midwest Biomedical Research Foundation-KCVAMC Affiliate, Kansas City, KS 64128, USA; 6 Pathology and Laboratory Medicine, The University of Kansas Medical Center, Kansas City, KS 66160, USA; otawfik@kumc.edu; 7 Molecular and Integrative Physiology, The University of Kansas Medical Center, Kansas City, KS 66160, USA; sgunewardena@kumc.edu; 8 Kidney Institute, The University of Kansas Medical Center, Kansas City, KS 66160, USA; 9 Sarah Cannon HCA Midwest Health Cancer Network, Overland Park, KS 66209, USA; 10 Hematology and Oncology, Kansas City Veterans Affairs Medical Center, Kansas City, MO 64128, USA; 11 School of Dentistry, University of Missouri-Kansas City, Kansas City, MO 64108, USA

**Keywords:** biomarkers, calcitriol, fibroblast growth factor 23, membrane vesicle biogenesis, microarray, inflammation, osteosarcoma, oxidative stress, Runx2

## Abstract

Osteosarcoma (OS) is an aggressive malignancy of bone affecting children, adolescents and young adults. Understanding vitamin D metabolism and vitamin D regulated genes in OS is an important aspect of vitamin D/cancer paradigm, and in evaluating vitamin D as adjuvant therapy for human OS. Vitamin D treatment of 143B OS cells induced significant and novel changes in the expression of genes that regulate: (a) inflammation and immunity; (b) formation of reactive oxygen species, metabolism of cyclic nucleotides, sterols, vitamins and mineral (calcium), quantity of gap junctions and skeletogenesis; (c) bone mineral density; and (d) cell viability of skeletal cells, aggregation of bone cancer cells and exocytosis of secretory vesicles. Ingenuity pathway analysis revealed significant reduction in Runx2 target genes such as fibroblast growth factor -1, -12 (*FGF1* and *FGF12*), bone morphogenetic factor-1 (*BMP1*), SWI/SNF related, matrix associated actin dependent regulator of chromatin subfamily a, member 4 (*SMARCA4*), Matrix extracellular phosphoglycoprotein (*MEPE*), Integrin, β4 (*ITGBP4*), Matrix Metalloproteinase -1, -28 (*MMP1* and *MMP28*), and signal transducer and activator of transcription-4 (*STAT4*) in vitamin D treated 143B OS cells. These genes interact with the inflammation, oxidative stress and membrane vesicle biogenesis gene networks. Vitamin D not only inhibited the expression of Runx2 target genes *MMP1*, *MMP28* and kallikrein related peptidase-7 (*KLK7*), but also migration and invasion of 143B OS cells. Vitamin D regulated Runx2 target genes or their products represent potential therapeutic targets and laboratory biomarkers for applications in translational oncology.

## 1. Introduction

Osteosarcoma (OS) is the primary malignancy of bone affecting children, adolescents and young adults and accounts for 20%–45% of all bone tumors. In the United States, about 500–1000 new cases are diagnosed annually. Development of lung metastasis is the main cause of death in OS patients [1,2,3]. With the advent of adjuvant chemotherapy, the five-year survival rate is approximately 70%. Despite aggressive chemotherapy and surgical treatments, one third of patients usually relapse with pulmonary metastases [4,5,6]. Several reports indicate that OS patients have decreased bone density, aberrations in vitamin D regulatory system, sub optimal vitamin D levels, oncogenic osteomalacia and increased incidence of pathological fractures which tend to increase lung metastases [7,8,9,10,11,12]. There is an unmet need to identify novel disease and/or therapeutic biomarkers for applications in laboratory medicine and translational oncology to stratify OS patients for response to therapy and enhance their survival.

Data from cellular, preclinical and epidemiological studies support the role of vitamin D in cancer chemoprevention/therapy, and clearly explain why and how vitamin D can affect tumor growth and proliferation, and that higher serum levels of 25(OH)D_3_ correlate to better survival and response to therapy [13]. 1α,25(OH)_2_D_3_ is the biologically active form of vitamin D and functions as a ligand for vitamin D receptors. Expression and activities of vitamin D metabolizing enzymes namely 1-α(OH)ase (encoded by gene *CYP27B1*) and 24 hydroxylase (encoded by gene *CYP24A1*) help in the regulation of steady state levels of 1α,25(OH)_2_D_3_. Different types of cancers have altered expression and activities of genes encoding vitamin D metabolizing enzymes or vitamin D modulators such as Fibroblast growth factor-23 (FGF23) [14,15,16,17,18,19,20,21,22,23,24,25,26]. FGF23 exerts autocrine effects on the proliferation of tumor cells as several solid tumor cells express FGF receptors [26]. FGF23 exerts a highly regulated feedback control on 1α,25(OH)_2_D_3_ mediated functions [27]. Presence of excess of FGF23 and matrix extracellular phosphoglycoprotein (MEPE) in oncogenic osteomalacia indicates similarities with genetically inherited rickets such as X-linked and autosomal dominant hypophosphatemic rickets [28]. In the bone microenvironment, it is the osteocytes which express FGF23, Dentin matrix protein-1 (DMP1) or phosphate regulating endopeptidase homolog, X-linked (Phex) [29]. Interestingly, a recent study suggests osteocyte as the cell of origin for osteosarcomagenesis [30]. In that study, the authors report abundant expression of DMP1 in murine, canine and human OS and evidence of osteoblastic/osteolytic lesions in mice injected with MLO-Y4 mouse osteocyte-like cell line [30]. Another study reported that FGF23 up regulates DMP1 mRNA in MLOY4 cells [31]. Neither the source and status of FGF23 nor its impact on oncogenic osteomalacia in OS is clear.

Runx2 is a transcription factor important for osteogenic differentiation and normal skeletal development. Recent studies highlight the role of Runx2 as a reliable OS biomarker for evaluating disease status and/or therapeutic response as there is high incidence of Runx2 genomic amplification and increased expression of Runx2 mRNA and protein in OS biopsy samples, tumor tissues from OS-mouse models, and its positive correlation with chemoresistance [32,33,34]. The role of vitamin D in regulating Runx2 expression and activity is not clear. Some studies indicate that Runx2 expression and activity is dependent on the expression levels of vitamin D receptor (VDR) and the differentiation status of the cell [32,33]. Another study reports that cholecalciferol (dietary vitamin D) modulates Runx2–DNA interactions and preferentially inhibits proliferation of breast cancer, and endothelial and bone cells [34]. All the above studies led us to investigate the role of 1,25(OH)_2_D_3_ in inhibiting expression of Runx2 and Runx2 target genes in 143B OS cells.

The molecular mechanisms underlying the antineoplastic properties of 1α,25(OH)_2_D_3_ are mainly mediated by modulation of expression of genes that regulate cellular proliferation, differentiation, apoptosis, angiogenesis, and oxidative stress [35,36]. We and others have previously reported that 1α,25(OH)_2_D_3_ exerts its antineoplastic effect by inducing differentiation and apoptosis of cancer cells [35,37]. The role of vitamin D and vitamin D regulatory system in OS is not clear and needs in depth genomic and proteomic investigational studies.

The main goal of this study is to evaluate 1α,25(OH)_2_D_3_ regulated gene expression in a metastatic human osteosarcoma cell line, 143B, at different stages of their growth by microarray gene expression profiling. The reason for choosing 143B cell line for our study is that these cells are extremely aggressive and show evidence of pulmonary metastasis when injected in vivo. Using this cell line, we have generated a pre-clinical bioluminescent osteosarcoma orthotopic mouse (BOOM) model [38]. It is our hypothesis that a number of 1α,25(OH)_2_D_3_ regulated genes are differentially expressed during proliferation, post-proliferation, and differentiation of 143B human OS cell line, and regulate cell cycle, cellular growth, proliferation and development, cell death, cell–cell and cell–matrix interactions, and cellular function and oxidative stress. To test the proposed hypothesis, we have compared vitamin D mediated changes in the expression of Runx2, Runx2 target genes and vitamin D regulatory system (*VDR*, *CYP27B1* and *CYP24A1*) at specific time points i.e., day 3, 9, and 15 as these match with the designated growth stages proliferation, post-proliferation, and differentiation, respectively, based on the results obtained from previous studies [32,33,34,37,39]. Knowledge gained from this study is innovative and significant, as it will identify key vitamin D target genes impacting potential cancer pathway signatures, and novel diagnostic biomarkers and will provide foundation for validating mechanism(s) underlying antineoplastic effects of vitamin D in the preclinical BOOM model [38].

## 2. Results

### 2.1. 1α,25(OH)_2_D_3_ Induces Stage-Specific Expression of Target Genes in 143B Human OS Cells

**Figure 1:**
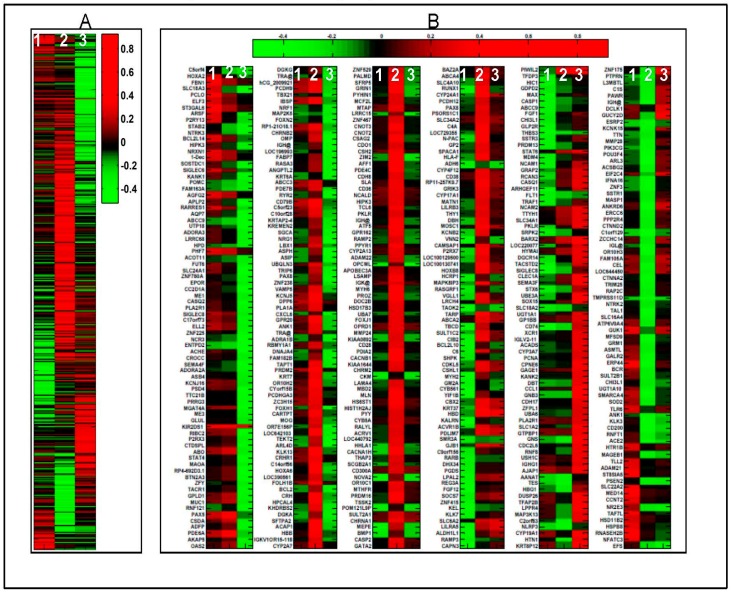
Heat map of Vitamin D target genes in 143B Osteosarcoma (OS) cells. Heat map of 1α,25(OH)_2_D_3_ induced gene expression fold changes (**A**) along with the names of the vitamin D-target genes (**B**) in 143B human OS cells during proliferation, post-proliferation, and differentiation relative to control (vehicle). Color bar represents log fold change values, red indicates up-regulated while green represents down regulated genes. Numbers 1–3 represents different growth stages of 143B cells post vitamin D treatment vs. control (ethanol or vehicle treated), namely proliferation, three days; post proliferation, nine days; and differentiation, 14 days. The colors red and green indicate genes that are up and down regulated, respectively.

**Figure 2:**
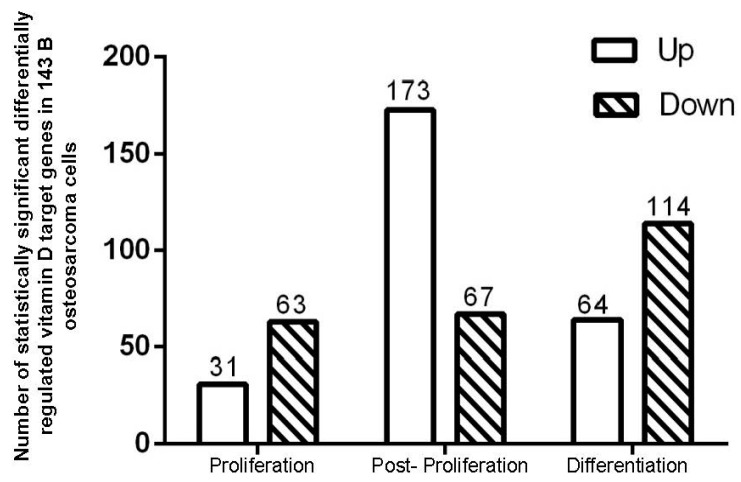
Comparison of number of statistically significant 1α,25(OH)_2_D_3_ induced target genes in 143B human OS cells during proliferation, post-proliferation, and differentiation relative to control (vehicle).

Microarray analysis of 143B OS cells treated with control vehicle (0.01% ethanol) or 100 nM 1α,25(OH)_2_D_3_ for 3, 9 and 15 days revealed a total of 500 differentially expressed genes (Figure 1 and Figure 2). Notably, 94 statistically significant (*p* < 0.05) (fold changes > 1.2) target genes including 31 up regulated and 63 down regulated genes in the proliferation group; a total of 240 statistically significant target genes including 173 up regulated and 67 down regulated genes in the post-proliferation group; and a total of 178 statistically significant target genes including 64 up regulated and 114 down regulated genes in the differentiation group were modified in the vitamin D treated relative to vehicle treated groups (Figure 2). The genes whose expression levels were most significantly changed by 1α,25(OH)_2_D_3_ in 143B and relevant to bone biology and bone tumor microenvironment specifically regulate: (a) inflammation and immunity; (b) formation of reactive oxygen species, metabolism of cyclic nucleotides, sterols, vitamins and calcium, quantity of gap junctions and skeletogenesis; and (c) bone mineral density, cell viability of skeletal cells, aggregation of bone cancer cells and exocytosis of secretory vesicles (Table 1).

**Figure 3:**
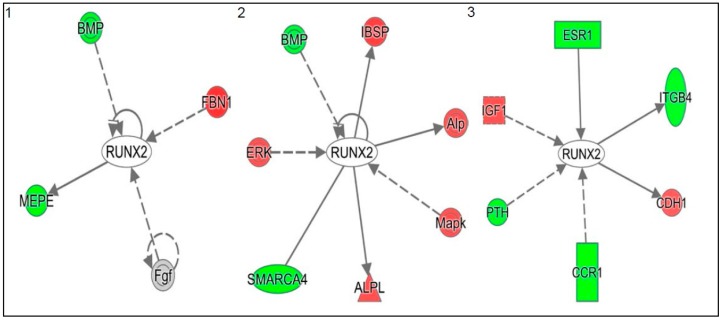
Vitamin D–Runx2 interactome reveals key genes that represent potential disease and therapeutic biomarkers in 143B human OS cells. Numbers 1–3 represents different growth stages of 143B cells post vitamin D treatment vs. control (ethanol or vehicle treated), namely proliferation, three days; post proliferation, nine days; and differentiation, 14 days. The colors red and green indicate genes that are up and down regulated, respectively. Solid lines imply direct interaction while dotted lines represent indirect interaction.

**Figure 4:**
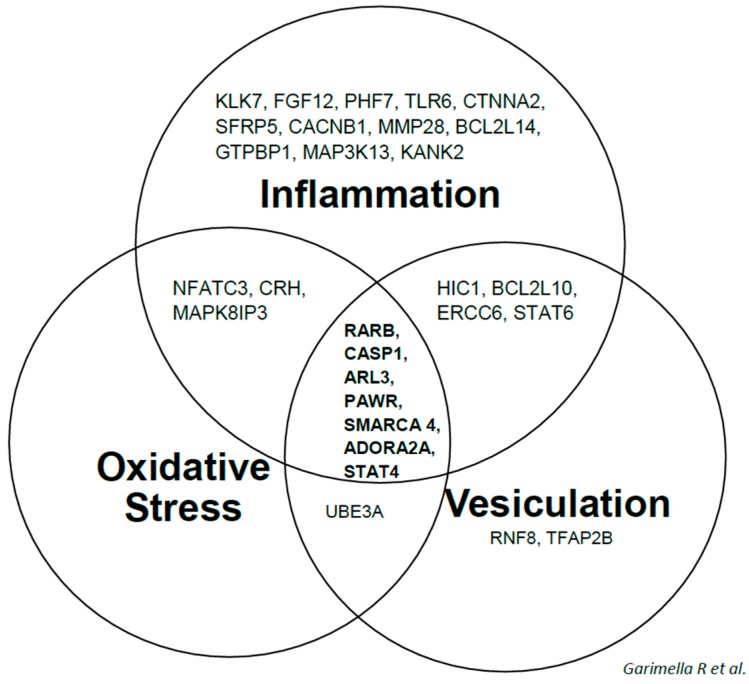
Venn diagram showing the most significant 1α,25(OH)_2_D_3_ regulated genes which interact with IPA constructed inflammation, oxidative stress and vesiculation networks of key molecules that were expressed during proliferation, post-proliferation and differentiation of 143B OS cell line.

Table 2 shows a list of top five biological functions (ranked by their statistical significance) of 1α,25(OH)_2_D_3_ regulated genes during proliferation, post-proliferation and differentiation growth stages of 143B cells. From the list of top ten genes differentially regulated in vitamin D treated 143B cells vs. vehicle treated 143B cells during proliferation, post-proliferation and differentiation, it is obvious that 1α,25(OH)_2_D_3_ modulated genes have functions that have either biological or clinical relevance as biomarkers for evaluating disease progression, diagnosis, prognosis and/or efficacy (Supplementary Tables; ST1A-F). These genes include kallikrein related peptidases-3 and -7 (*KLK3* and *KLK7*), a disintegrin and metallopeptidase domain 21 (*ADAM21*), hypermethylated in cancer (*HIC1*), retinoic acid receptor beta (*RARB*), secreted frizzled receptor 5 (*sFRP5*), corticotropin releasing hormone (*CRH*), PRKC apoptosis WT1 regulator (*PAWR*), and adenosine A2a receptor (*ADORA2A*). Ingenuity system pathway analyses revealed a number of vitamin D down regulated expression of Runx2 modulators or Runx2 target oncogenes such as *FGF1*, *FGF12*, bone morphogenetic protein 1 (*BMP1*), *MEPE*, SWI/SNF related, matrix associated actin dependent regulator of chromatin subfamily a, member 4 (*SMARCA4*), parathyroid hormone (*PTH*), estrogen receptor 1 (*ESR1*), and chemokine (C–C) motif receptor 1 (*CCR1*) which either directly enhance neoplastic properties and/or interact with the inflammation, oxidative stress and membrane vesicle biogenesis genetic networks and modulate tumor microenvironments [40,41] (Figure 3 and Figure 4; Figure S1A–C (inflammation); Figure S2A–C (oxidative stress); and Figure S3A–C (vesiculation)).

### 2.2. Real Time Quantitative Polymerase Chain Reaction, Western Blotting, and Immunohistochemistry Detects Vitamin D Target Genes in 143B Cells and Human OS Tissue Microarrays

**Figure 5:**
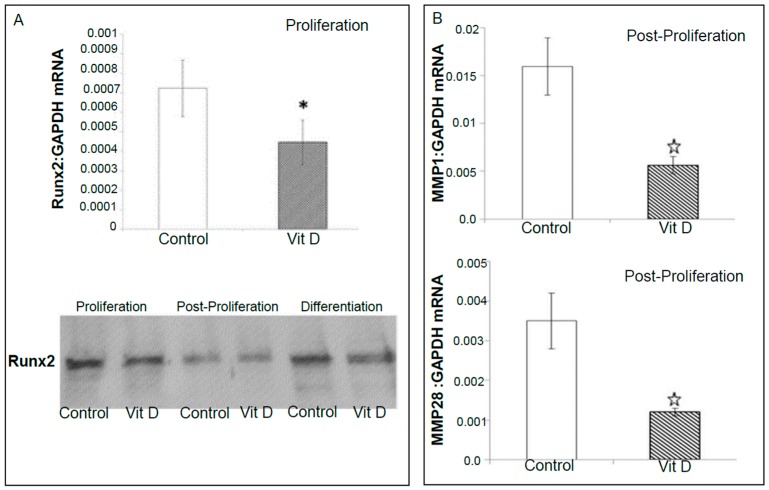
Effect of vitamin D in inhibiting the gene expression of: Runx2 (**A**); and Runx2 target genes MMP1 and MMP28 (**B**), in 143B osteosarcoma cells. Star indicates statistical significance of *p* ≤ 0.05.

**Figure 6:**
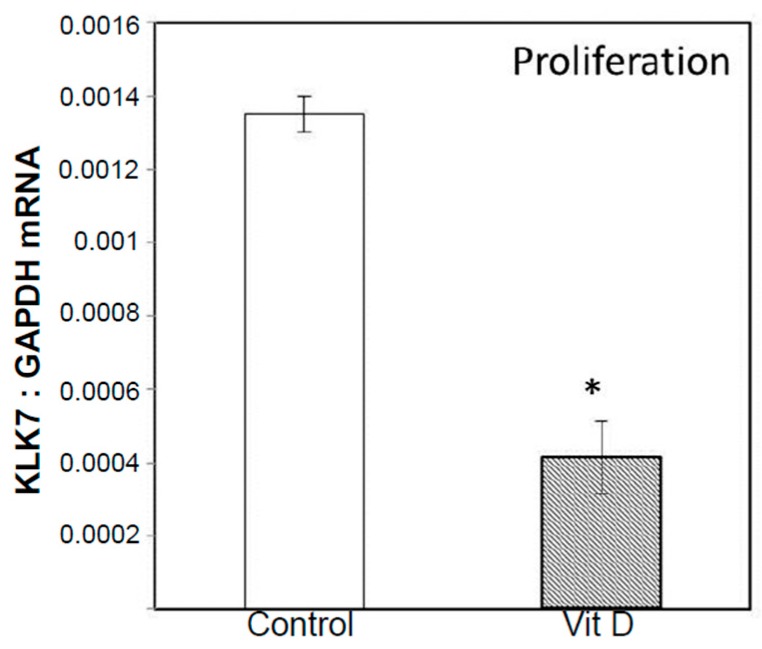
Detection of reduced KLK7 mRNA expression in 1α,25(OH)_2_D_3_ treated 143B cells by RT-qPCR. Asterisk represents statistical significance of *p* ≤ 0.05.

**Figure 7:**
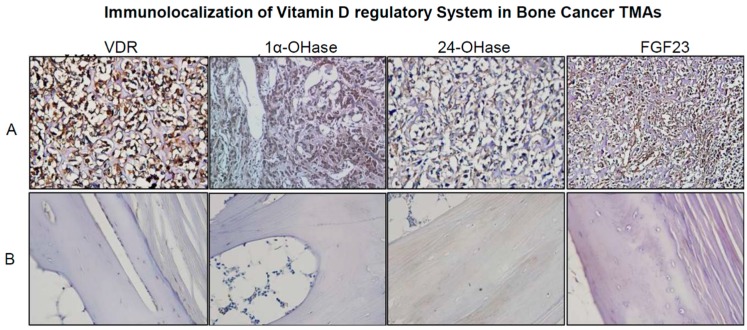
Immunodetection of vitamin D regulatory system comprised of VDR, 1α-OHase, 24-OHase and FGF23 protein in the osteoblastic core (**A**); versus control or healthy bone tissue core (**B**) of commercially available osteosarcoma tissue microarray. Original magnification, 40×.

Microarray data showed an increased expression of CYP24 mRNA in 1α,25(OH)_2_D_3_ treated 143B cells during post-proliferation, which was confirmed by RT-qPCR. The changes in the relative expression of *CYP24*, *CYP27B1*, and *VDR* in 1α,25(OH)_2_D_3_ treated vs. untreated 143B OS cell line were not however significant at the experimentally tested time points (Figure S4). This was mainly due to the time points selected in the study (Day 3, 9 and 15) as previous studies indicate maximal changes in the gene expression (especially for CYP24) within the 24 h [42]. The expression of vitamin D target genes (*CYP24*, *CYP27B1*, and *VDR*) at the protein level was detected by Western blotting, which confirmed qPCR results (Figure S4). RT-qPCR studies demonstrated inhibition of expression of Runx2 (proliferation) and Runx2 target genes matrix metalloproteinases, *MMP1* (post-proliferation) and *MMP28* (post-proliferation) in the same samples which were used for microarray profiling studies (Figure 5). Vitamin D mediated down regulation of MMP 28 (Figure 5) and KLK7 (proliferation) (a MMP processing protease (Figure 6)) expression by RT-qPCR confirms microarray results (Table 2A and Table S2A,C). Interestingly, osteoblastic OS core group of bone cancer tissue microarray (TMA) displayed intense expression of VRS compared to fibroblastic and talangiectactic OS (Figure 7) but the expression varied with tumor site (Figure S5). Increased expression of VDR and FGF23 relative to other VRS components is interesting, especially in the context of increased Runx2 expression as previously observed in the same OS-core type, and also in the tumor tissue isolated from the BOOM model [38]. The immunostaining of VRS in the disease free healthy bone was very weak or absent compared to the tumor tissue.

### 2.3. 1α,25(OH)_2_D_3_ Inhibits Migration and Invasion of 143 Cells

**Figure 8:**
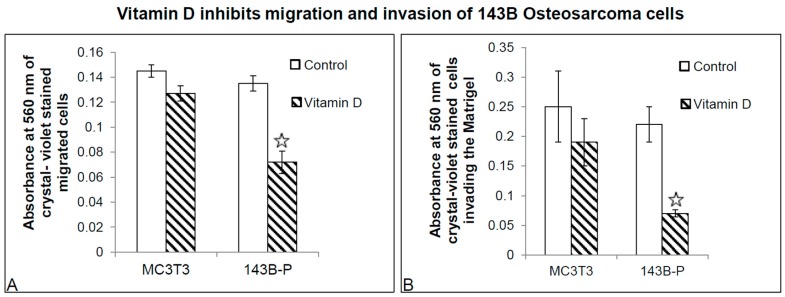
Comparative effect of 1α,25(OH)_2_D_3_ on migration and invasion of 143B human OS cells vs. MC3T3, pre-osteoblast cells. Migration (**A**); and invasion (**B**) of 143B (P denotes parental 143B human OS cell line as obtained from ATCC) and MC3T3 cells was evaluated by quantifying crystal violet staining of migrated/invaded cells through the membranes in transwell chamber assays by monitoring a change in the absorbance at 560 nm. Star indicates statistical significance * *p* ≤ 0.05.

Calcitriol or 1α,25(OH)_2_D_3_ significantly inhibited not only the migration of 143B cells, but also their invasion through Matrigel (Figure 8). This observation is consistent with the real time qPCR data showing decreased Runx2, MMP1 and MMP28 gene expression in 1α,25(OH)_2_D_3_ treated 143B cells, and microarray data which revealed that 1α,25(OH)_2_D_3_ regulated the expression of a number of genes involved in cellular movement, for example, *ADAM21*, *MMP28*, and adherens junction associated protein 1 (*AJAP1*) (Table S1B,C,F).

## 3. Discussion

In recent years, there has been a burgeoning interest in identifying biomarkers/molecular signatures, which have great potential to revolutionize genomic and/or personalized medicine. Advances in molecular technologies allow the application of biomarkers as an invaluable cost-effective detection tool either used alone or in combination with existing imaging methods for early screening or risk assessment, detection and diagnosis, and developing effective cancer therapies. This study provides a novel and valuable insight into global gene expression profiling of 1α,25(OH)_2_D_3_-mediated-growth stage specific changes in 143B, a metastatic OS cell line.

Identification of 1α,25(OH)_2_D_3_ regulated genes such as *RARB*, *CASP1*, *ARL3*, *PAWR*, *SMARCA4*, *ADORA2A* and *STAT4* and their interaction with the inflammation, oxidative stress and membrane vesicle biogenesis gene networks is very interesting. Most of the genes constituting this panel serve as biomarkers for efficacy, prognosis, diagnosis, disease progression (targeting inflammation, oxidative stress and vesiculation gene networks) and response to therapy [43,44,45,46,47,48,49,50,51,52,53,54,55,56,57]. Vitamin D mediated genetic networks such as those identified in our study, for example inflammation, oxidative stress and membrane vesicle biogenesis pathways in 143B cells, shed insight into the mechanism(s) underlying antineoplastic effects of vitamin D in human OS.

Immunodetection of VRS especially VDR and FGF23 in osteoblastic core of OS-TMAs is interesting as previous studies have reported tumor promoting effects of FGF23 either directly or indirectly by affecting the bioavailability and catabolism of 1α,25(OH)_2_D_3_ which in turn is important for mediating antineoplastic functions [26,58]. FGF/FGFR signaling increases the transcriptional activity of Runx2, an osteogenic transcription factor [59], which is overexpressed in several cancers including osteosarcoma [60]. Mice overexpressing FGF23 display increased expression of Runx2 and alkaline phosphatase, receptor activator of NFκB ligand or RANKL and osteoprotegerin transcripts, MMP9 and cathepsin K immunoexpression along with increased serum concentrations of C terminal telopeptide of collagen (CTX) with increased bone resorptive activity [61]. Vitamin D mediated down regulation of FGF1 in OS cells is significant as previous studies have detected increased levels of serum FGF1 and FGFR1 amplification in OS patients [62,63]. FGF1 mediated activation of PKA and PKC signaling pathways induces nucleoside triphosphate pyrophosphohydrolase (NTPPPH) expression in OS cells [64]. Both FGF1 and FGF2 stimulate FGF23 transcriptional activity, in OS cells, which in turn was blocked in the presence of FGF1 inhibitor, PD173704 [65]. Future studies will investigate the role of FGF23 in stimulating expression of Runx2 and Runx2 target genes and enhancing transcriptional activity of Runx2 leading to increased biogenesis of tumor supportive EMVs that drive the vicious cycle and contribute to vitamin D deficiency. We and others have shown that NTTPPH overexpression results in osteomalacia in long bones, and is localized in EMVs [66,67]. We have previously reported the role of calcium influx and cAMP signaling in EMV biogenesis in OS cells [68]. Whether FGF1 stimulates EMV biogenesis in OS via calcium or cAMP/PKA signaling in OS cells is unknown. Vitamin D mediated down regulation of FGF12 mRNA expression in microarray gene expression studies supports the neoplastic role of FGFs in osteosarcoma pathobiology as it stimulates proliferation, extra cellular matrix remodeling, inflammation, and angiogenesis. CCR1 is important for migration and invasion of osteosarcoma cells. Overexpression of FGFR3 in MM cells stimulates secretion of CCL3, a ligand for CCR1 and activates RAS-MAPK pathway. Inactivation of CCR1 suppresses not only cancer cells but also cells in the bone tumor microenvironment reducing the overall osteolytic tumor burden [69]. Whether FGFs stimulate FGFR mediated CCL3/CCR1 signaling and its downstream effects on Runx2 target genes such as *RANKL*, *MMPs*, parathyroid hormone related peptide (*PTHrP*), survivin, and vascular endothelial growth factor (*VEGF*) are unknown in OS. The role of FGF ligands (FGF1, FGF12 or FGF23) or receptors (FGFR1) as biomarkers of OS disease progression or therapeutic response seems promising for stratification of patients to improve survival outcomes.

Vitamin D regulation of Runx2 gene interactions in our study highlights the role of Runx2 and Runx2 target genes in OS pathobiology. Previous studies report that Runx2 increases the expression of prometastatic Runx2 target genes like integrins, focal adhesion kinase *FAK*/*PTK2* or Talin (*TLN*), *MMPs*, *PTHrP*, *VEGF*, bone sialoprotein (*BSP*), osteopontin, survivin, etc. [70,71,72,73,74,75,76,77]. Runx2 expression has a pro-survival role in rapidly proliferating tumor cells in the bone microenvironment by promoting PTH or PTHrP mediated antiapoptototic effect and inducing the expression of survivin [78]. In OS, a number of growth factors such as BMPs, PTH/PTHrP, TGF-β, and FGF23 activate Runx2 either directly or indirectly and promoting Runx2 phosphorylation. Runx2 gene product PTHrP can activate PTHrP/PTHrPR1 signaling and raise intracellular cyclic-adenosine monophosphate cAMP and calcium levels which in turn results in cytoskeleton changes and potentially lead to exosomal biogenesis. Vitamin D mediated down regulation of Runx2 target gene integrin, β4 (*ITGB4*) is interesting as this integrin regulates the expression and function of ezrin, an important biomarker and mediator of OS pulmonary metastasis [79]. Detection of ITGB4 in the cancer exosomes and its role in metastatic organotropism, especially lung tropism, opens up an important question whether vitamin D mediated inhibition of *ITGB4* impacts pulmonary metastasis via reduced amounts in exosomes/EMVs derived from OS cells and inhibition of exosomal uptake by lung fibroblasts [80]. Recently, a novel role of Runx2 in tumor cell survival became evident as studies reveal that Runx2 inhibited the apoptotic pathway by activating the expression of survivin and Bcl2 [81]. Survivin is an important cancer biomarker of OS and its expression correlates well with relapse and chemoresistance [82,83]. Runx2 overexpression leads to osteopenia and multiple fractures, through increased receptor activator of NF-κB ligand (RANKL) expression which in turn could stimulate OCL activity [84,85]. Vitamin D mediated down regulation of expression of Runx2 and Runx2 target genes such as MMP1, MMP28 is important as Runx2 expression is associated with poor chemotherapy response in OS [86]. Elegant genomic occupancy and chromatin immunoprecipitation (ChIP) studies reveal that in OS cell lines, Runx2 regulates the functions of genes of focal adhesion pathway, which regulate cell motility and adhesion (*TLN1* and *FAK*) [77].

To further validate the biological and therapeutic relevance of vitamin D in inhibiting expression and/or activity of Runx2 target genes in OS, future studies will include: (a) determining the effect of vitamin D in Runx2 overexpressing or Runx2 siRNA treated OS cell lines differing in their p53 and/or ki-ras status (U2OS, SaOS2, HOS and 143B) and that display differential neoplastic activity such as aggressiveness (migration and invasion), angiogenesis, EMV biogenesis, metastasis and chemoresistance; (b) evaluating the role of vitamin D in modulating differential expression of miRNAs in OS cell lines; and (c) elucidating the role of vitamin D in inhibiting Runx2 mediated osteosarcoma bone disease and metastases in vivo, using the BOOM model.

## 4. Materials and Methods

### 4.1. Cell Culture

Osteosarcoma cell line 143B was obtained from American Type Culture Collection (Manassas, VA, USA). We have validated and established the oncogenic activity of 143B OS cells in the BOOM model [38]. The cells were cultured in Dulbecco’s Modified Eagle Medium (DMEM), supplemented with 100 U/mL Penicillin, and 100 µg/mL Streptomycin, 10% Fetal Bovine Serum, and 1% non-essential amino acids under an atmosphere of 5% CO_2_ at 37 °C in a humidified incubator. Cells were seeded at a density of 0.5 × 10^4^ cells per well in a 6-well tissue-culture plate. 143B cells were treated with control vehicle (0.01% ethanol) or vitamin D or 1α,25(OH)_2_D_3_ (100 nM) and medium was changed every other day. To stimulate differentiation, L-ascorbic acid 2-phosphate (50 µg/mL) and β-glycerophosphate (5 mM) were added to the cultures. Treatment sets were repeated for three different experiments.

### 4.2. RNA Isolation and Assessment of RNA Quality and Purity

Total RNA was isolated from 143B OS cells that were at different stages of growth, i.e., proliferative (72 h), post-proliferative (9 days) and differentiation (15 days), using RNeasy Mini Kit (Qiagen, Santa Clara, CA, USA). The quality and purity of isolated RNA was evaluated by Agilent bioanalyzer and only those samples with high values for RNA integrity number (RIN) were selected for hybridization studies (Table S2 and Figure S6).

### 4.3. Microarray Data Analysis

Preparation of cRNA targets was done using standard Affymetrix protocols. The cRNA fragments were allowed to hybridize to the sequences on the chip of Human Genome U133A 2.0 arrays. This array consists of ~18,400 transcripts representing over 14,500 genes. The probe intensity values were corrected for background noise, and subsequently normalized and summarized using Robust Multi-array Average procedure [87]. The resulting log (base 2) intensity values were used for differential expression calculations. Fold change and *p*-values were calculated for genes that were differentially expressed in vitamin D treated proliferation, post-proliferation and differentiation vs. control (vehicle treated) groups. Fold change statistics for individual genes were derived based on previously published statistical methods [88,89]. Each treatment and control group consisted of biological triplicates for analysis. Genes with an absolute fold-change greater than or equal to 1.2 and having a *p*-value less than or equal to 0.05 were considered significantly regulated. All computations were performed in Matlab (R2012b, The MathWorks Inc., and Natick, MA, USA) and the Partek Genomic suite (v 6.5, Partek Inc., St. Louis, MO, USA). Biological functions, pathways and upstream regulators associated with significantly perturbed genes were identified using the Ingenuity Pathways Analysis software (IPA, Ingenuity Systems, available online: www.Ingenuity.com). IPA identifies significant molecular networks, biological functions and upstream regulators associated with a set of genes based on information gathered in the Ingenuity Pathway Knowledge Base (IPKB).

### 4.4. Validation of Selected Vitamin D Regulated Target Genes by Real-Time Quantitative Polymerase Chain Reaction (RT-qPCR) and Western Blotting in 143B Human Osteosarcoma Cell Line

For detection and validation of selected vitamin D regulated target genes, total RNA from 143B (±1α,25(OH)_2_D_3_) was isolated and probed with primers for VRS comprising of VDR, CYP27B1, CYP24, Runx2 and Runx2 target genes (*MMP-1* and *MMP28*). (Primer sequences and PCR cycling conditions are provided as supplementary information). Real time qPCR was performed according to the standard protocol recommended by Applied Biosystems 7500 Sequence Detection system and software (Applied Biosystems, Foster City, CA, USA), and iCycler (Bio-Rad, Hercules, CA, USA). Relative quantitation of target mRNA expression, normalized to an endogenous control and relative to a calibrator (osteoblast RNA) was calculated using the mathematical expression for fold change, i.e., 2^−ΔΔ*C*t^ (fold), as described by Livak et al, where Δ*C*_t_ = *C*_t_ of the target gene –*C*_t_ of the endogenous control gene (GAPDH), and ΔΔ*C*_t_ = Δ*C*_t_ of the samples for target gene –Δ*C*_t_ of the calibrator for the target gene [90].

For the detection of expression of VDR, 1-α OHase, 24-hydroxylase and Runx2 proteins, Western blot analyses was performed. Twenty-five to fifty micrograms of crude cell-lysate (protein) was solubilized in SDS-sample buffer, electrophoresed on 12% denaturing polyacrylamide gels and visualized by Comassie blue stain. For immunoblotting, the proteins from the gel was transferred on to a PVDF membrane and incubated with following VRS primary antibodies: anti-VDR, anti-1-α OHase, anti-24-hydroxylase, and anti-Runx2. The primary antibodies for VRS regulatory system were purchased from Santa Cruz Biotechnology Inc. (Santa Cruz, CA, USA) and used at a concentration of 1:200. The immunostained bands were visualized using an ECL chemiluminescent detection system (Amersham Biosciences, Piscataway, NJ, USA). Extracts of breast cancer cells MCF7 and kidney tissue were used as positive control [39].

### 4.5. Detection and Immunolocalization of VRS in Human OS Tissue Microarrays

A bone cancer tissue arrays containing at least 6 cases of OS (osteoblastic, parosteal, fibroblastic, talangiectatic, conventional OS of left lower limb, and proximal humerus) in duplicates, and 2 cores of disease-free healthy bone tissue per array were purchased from US Biomax (Rockville, MD, USA). All the OS cores were classified as malignant, stage II b, and T2N0M0 grade. Immunohistochemistry for markers of vitamin D regulatory system, i.e., VDR, 1-α hydroxylase and 24-hydroxylase was performed as described below. Briefly, the arrays were fixed in 4% paraformaldehyde and standard immunostaining procedures was performed using the ABC staining kit from Santa Cruz Biotechnology Inc. Primary antibodies for VDR, 1-α hydroxylase, 24-hydroxylase, FGF23 were purchased from Santa Cruz Biotechnology Inc. All the primary antibodies were used at 1:200 dilutions. Immunostaining intensity in the OS cores was compared to the disease free bone tissue cores or the control group. As negative control, primary antibody was excluded in the immunostaining.

### 4.6. Statistical Analysis

For quantitation of real time qPCR data, excel software was used to calculate mean and standard errors of means, and t test was used to analyze differences in 1α,25(OH)_2_D_3_ vs. untreated samples. A *p* value of <0.05 was considered as statistically significant. For analysis of microarray data, statistical methods are described in the microarray data analysis sub-section.

## 5. Conclusions

In conclusion, our data highlights the role of vitamin D in targeting Runx2 pathway in 143B OS cell line, specifically in the inhibition of genes critical for cell cycle, cellular proliferation, survival, migration and invasion, cell–cell and cell–matrix interactions, microtubule dynamics and cytoskeletal rearrangements, EMV biogenesis and chemoresistance. This study suggests a novel role of vitamin D regulated Runx2 target genes/or their products as clinically relevant biomarkers in vitamin D mediated chemoprevention strategies or in adjuvant therapy for OS disease management.

## Figures and Tables

**Table 1:** Ingenuity pathway analysis (IPA) ranked vitamin D modulated biofunctions relevant to bone biology and bone tumor microenvironment.

Stage	Disease or Function	*p* Value	Molecules
Proliferation	formation of bone cells	9.69 × 10^−3^	TSHR
	metabolic bone disease	2.63 × 10^−2^	BMP1 and RGN
	egression of natural killer cells; non-canonical wnt signaling	4.85 × 10^−3^	RORC
	inflammation	4.85 × 10^−3^	ITGAM
		9.69 × 10^−3^	FGF1
	cellular assembly and vesicle trafficking	4.85 × 10^−3^	RAB7A
Post-proliferation	Formation of reactive oxygen species	2.48 × 10^−3^	APOE, CD28, GRIN1, P2RX7, PIK3CG, SOD2 (activation *z* score 1.66)
	metabolism of cyclic nucleotides	1.15 × 10^−4^	APOE, CASP2, CHRM2, CRH, CRHR1, GALR2, GRM1, NPY4R, OPRD1, PDE4C, PDIA2, PIK3CG, PYY, RAMP2 (activation *z* score: 1.66)
	catabolism of sterol	2.52 × 10^−4^	APOE, CEL, CYP24A1
	quantity of gap junctions	5.88 × 10^−3^	APOE, GJB1, GRIN1, PCDHGA3 (activation *z* score: 1.73)
	vitamin and mineral metabolism (quantity of calcium ions)	9.39 × 10^−3^	APOE, CACNA1H, CD28, CD38, CHRM2, CRH, GRIN1, GRM1, IBSP, MLN, P2RX7, PIK3CG, PSEN2, PYY, THY1 (activation *z* score 2.6)
	Deformation of bone	1.42 × 10^−2^	HBB, PAX8
Differentiation	Bone mineral density	1.80 × 10^−5^	DCN, ESR1, IGF1, PRLR, PTH, RGN
	cell viability of bone cell lines	5.83 × 10^−3^	PTH
	aggregation of bone cancer cells	1.16 × 10^−2^	CDH1
	exocytosis of secretory vesicles	1.16 × 10^−2^	IGF1

**Table 2:** Ingenuity pathway analysis (IPA) ranked vitamin D mediated changes in top five biofunctions (based on their *p* values) in 143B osteosarcoma cells.

Category	Diseases or Functions Annotation	*p* Value	Molecules
**Proliferation**			
Cancer	thyroid cancer	6.10 × 10^−5^	FLT1, GDF15, KLK10, KLK7, RARB, TSHR
Endocrine System Disorders	thyroid cancer	6.10 × 10^−5^	FLT1, GDF15, KLK10, KLK7, RARB, TSHR
Cell-To-Cell Signaling and Interaction	communication of cells	2.05 × 10^−4^	ACVR1B, CAPN3, CASP1, FGF12, FLT1, GDF15, ITGAM, PAK2, RAMP3, RARB, RASGRF1, RORC, SMAD5-AS1, TACSTD2, TLR6, TSHR
Cellular Movement	cell movement of prostate cancer cell lines	4.68 × 10^−4^	CTSZ, GDF15, HIC1, PAK2 (activation z score: 1.97)
Cell-To-Cell Signaling and Interaction	signal transduction	5.35 × 10^−4^	ACVR1B, CAPN3, CASP1, FGF12, FLT1, GDF15, PAK2, RAMP3, RARB, RASGRF1, RORC, SMAD5, -AS1, TACSTD2, TLR6, TSHR
**Post-Proliferation**			
Behavior	behavior	2.31 × 10^−5^	ABCA2, APOE, BCR, CACNB1, CARTPT, CD36, CDKL5, CDO1, CHRM2, CRH, CRHR1, CTNNA2, CTNND2, DBH, ERCC6, GALR2, GATA2, GRIN1, GRM1, HBB, HOXB8, KCNJ5, LAMA4, LSAMP, MBD2, NPR3, NPY4R, NTRK2, OPRD1, P2RX7, PAWR, PSEN2, PTPRN, PYY, SOD2
Small Molecule Biochemistry	sulfation of raloxifene	9.22 × 10^−5^	SULT1C2, SULT2A1, SULT2B1
Neurological Disease	seizures	9.25 × 10^−5^	ADAM22, ANKRD6, ATP6V0A4, CACNA1H, CRH, DBH, GJB1, GPR162, GRIK3, GRIN1, GRM1, HBB, HBD, NTRK2, PSEN2, PTPRN, SLC4A10, SOD2, SSTR1
Cell Morphology	abnormal morphology of myelin sheath	1.13 × 10^−4^	ABCA2, APOE, ERCC6, GJB1, LAMA4
Nervous System Development and Function	abnormal morphology of myelin sheath	1.13 × 10^−4^	ABCA2, APOE, ERCC6, GJB1, LAMA4
**Differentiation**			
Tissue Development	development of mammary alveolus	7.10 × 10^−6^	CDH1, IGF1, PRLR, TGFA
Digestive System Development and Function	abnormal morphology of digestive system	8.20 × 10^−6^	ABCB11, CCR1, DCN, ESR1, GJB1, IKZF1, KRT6A, PRLR, RAD23B, RGN, SOSTDC1, STAT4, TGFA
Organ Development	response of liver	1.21 × 10^−5^	ABCB11, ADORA2A, CASP1, CXCL6, ESR1, IGF1, STAT4, STAT6, TGFA (activation Z score: 0.179)
Carbohydrate Metabolism	deposition of polysaccharide	1.55 × 10^−5^	ESR1, IGF1, PTH
Skeletal and Muscular System Development and Function	bone mineral density	1.80 × 10^−5^	DCN, ESR1, IGF1, PRLR, PTH, RGN

## Supplementary Materials

Supplementary materials can be found at www.mdpi.com/1422-0067/18/3/642/s1.

## Abbreviations

BMP	Bone morphogenetic protein
BOOM	Bioluminescent osteosarcoma orthotopic mouse
EMV	Extracellular membrane vesicles
FGF	Fibroblast growth factor
FGFR	Fibroblast growth factor receptor
ITGBP4	Integrin, β4
MEPE	Matrix extracellular phosphoglycoprotein
MMP	Matrix Metalloproteinase
OS	Osteosarcoma
PTHrP	Parathyroid hormone related peptide
Runx2	Runt-related transcription factor 2
SMARCA4	SWI/SNF related, matrix associated actin dependent regulator of chromatin subfamily a, member 4

## References

[1] Ferrari S., Palmerini E. (2007). Adjuvant and neoadjuvant combination chemotherapy for osteogenic sarcoma. Curr. Opin. Oncol..

[2] Bruland O.S., Pihl A. (1997). On the current management of osteosarcoma. A critical evaluation and a proposal for a modified treatment strategy. Eur. J. Cancer.

[3] Harris M.B., Gieser P., Goorin A.M., Ayala A., Shochat S.J., Ferguson W.S., Holbrook T., Link M.P. (1998). Treatment of metastatic osteosarcoma at diagnosis: A pediatric oncology group study. J. Clin. Oncol..

[4] Meyers P.A., Gorlick R. (1997). Osteosarcoma. Pediatr. Clin. N. Am.

[5] Link M.P. (1986). Adjuvant therapy in the treatment of osteosarcoma. Important Adv. Oncol..

[6] Longhi A., Errani C., de Paolis M., Mercuri M., Bacci G. (2006). Primary bone osteosarcoma in the pediatric age: State of the art. Cancer Treat. Rev..

[7] Pirker-Fruhauf U.M., Friesenbichler J., Urban E.C., Obermayer-Pietsch B., Leithner A. (2012). Osteoporosis in children and young adults: A late effect after chemotherapy for bone sarcoma. Clin. Orthop. Relat. Res..

[8] Bilariki K., Anagnostou E., Masse V., Elie C., Grill J., Valteau-Couanet D., Kalifa C., Doz F., Sainte-Rose C., Zerah M. (2010). Low bone mineral density and high incidences of fractures and vitamin D deficiency in 52 pediatric cancer survivors. Hormone Res. Paediatr..

[9] Ruza E., Sotillo E., Sierrasesumaga L., Azcona C., Patino-Garcia A. (2003). Analysis of polymorphisms of the vitamin D receptor, estrogen receptor, and collagen Iα1 genes and their relationship with height in children with bone cancer. J. Pediatr. Hematol. Oncol..

[10] Azcona C., Burghard E., Ruza E., Gimeno J., Sierrasesumaga L. (2003). Reduced bone mineralization in adolescent survivors of malignant bone tumors: Comparison of quantitative ultrasound and dual-energy X-ray absorptiometry. J. Pediatr. Hematol. Oncol..

[11] Lee R.K., Chu W.C., Leung J.H., Cheng F.W., Li C.K. (2012). Pathological fracture as the presenting feature in pediatric osteosarcoma. Pediatr. Blood Cancer.

[12] Lamont E.B., Cavaghan M.K., Brockstein B.E. (1999). Oncogenic osteomalacia as a harbinger of recurrent osteosarcoma. Sarcoma.

[13] Deeb K.K., Trump D.L., Johnson C.S. (2007). Vitamin D signalling pathways in cancer: Potential for anticancer therapeutics. Nat. Rev. Cancer.

[14] Mitschele T., Diesel B., Friedrich M., Meineke V., Maas R.M., Gartner B.C., Kamradt J., Meese E., Tilgen W., Reichrath J. (2004). Analysis of the vitamin D system in basal cell carcinomas (BCCs). Lab. Investig. J. Tech. Methods Pathol..

[15] Friedrich M., Diesing D., Cordes T., Fischer D., Becker S., Chen T.C., Flanagan J.N., Tangpricha V., Gherson I., Holick M.F. (2006). Analysis of 25-hydroxyvitamin D3-1α-hydroxylase in normal and malignant breast tissue. Anticancer Res..

[16] Friedrich M., Rafi L., Mitschele T., Tilgen W., Schmidt W., Reichrath J. (2003). Analysis of the vitamin D system in cervical carcinomas, breast cancer and ovarian cancer. Recent Results Cancer Res. Fortschritte Krebsforschung.

[17] Albertson D.G., Ylstra B., Segraves R., Collins C., Dairkee S.H., Kowbel D., Kuo W.L., Gray J.W., Pinkel D. (2000). Quantitative mapping of amplicon structure by array CGH identifies CYP24 as a candidate oncogene. Nat. Genet..

[18] Cross H.S., Bareis P., Hofer H., Bischof M.G., Bajna E., Kriwanek S., Bonner E., Peterlik M. (2001). 25-hydroxyvitamin D3-1α-hydroxylase and vitamin D receptor gene expression in human colonic mucosa is elevated during early cancerogenesis. Steroids.

[19] Cross H.S., Bises G., Lechner D., Manhardt T., Kallay E. (2005). The vitamin D endocrine system of the gut—Its possible role in colorectal cancer prevention. J. Steroid Biochem. Mol. Boil..

[20] Ogunkolade B.W., Boucher B.J., Fairclough P.D., Hitman G.A., Dorudi S., Jenkins P.J., Bustin S.A. (2002). Expression of 25-hydroxyvitamin D-1α-hydroxylase mRNA in individuals with colorectal cancer. Lancet.

[21] Anderson M.G., Nakane M., Ruan X., Kroeger P.E., Wu-Wong J.R. (2006). Expression of VDR and CYP24A1 mRNA in human tumors. Cancer Chemother. Pharmacol..

[22] Parise R.A., Egorin M.J., Kanterewicz B., Taimi M., Petkovich M., Lew A.M., Chuang S.S., Nichols M., El-Hefnawy T., Hershberger P.A. (2006). CYP24, the enzyme that catabolizes the antiproliferative agent vitamin D, is increased in lung cancer. Int. J Cancer.

[23] Mimori K., Tanaka Y., Yoshinaga K., Masuda T., Yamashita K., Okamoto M., Inoue H., Mori M. (2004). Clinical significance of the overexpression of the candidate oncogene CYP24 in esophageal cancer. Ann. Oncol..

[24] Schwartz G.G., Eads D., Rao A., Cramer S.D., Willingham M.C., Chen T.C., Jamieson D.P., Wang L., Burnstein K.L., Holick M.F. (2004). Pancreatic cancer cells express 25-hydroxyvitamin D-1α-hydroxylase and their proliferation is inhibited by the prohormone 25-hydroxyvitamin D3. Carcinogenesis.

[25] Reichrath J., Rafi L., Rech M., Mitschele T., Meineke V., Gartner B.C., Tilgen W., Holick M.F. (2004). Analysis of the vitamin D system in cutaneous squamous cell carcinomas. J. Cutan. Pathol..

[26] Feng S., Wang J., Zhang Y., Creighton C.J., Ittmann M. (2015). FGF23 promotes prostate cancer progression. Oncotarget.

[27] Razzaque M.S. (2012). FGF23, klotho and vitamin D interactions: What have we learned from in vivo mouse genetics studies?. Adv. Exp. Med. Boil..

[28] Rowe P.S. (2004). The wrickkened pathways of FGF23, MEPE and PHEX. Crit. Rev. Oral Biol. Med. Off. Publ. Am. Assoc. Oral Biol..

[29] Bonewald L.F. (2011). The amazing osteocyte. J. Bone Min. Res. Off. J. Am. Soc. Bone Min. Res..

[30] Sottnik J.L., Campbell B., Mehra R., Behbahani-Nejad O., Hall C.L., Keller E.T. (2014). Osteocytes serve as a progenitor cell of osteosarcoma. J. Cell. Biochem..

[31] Kyono A., Avishai N., Ouyang Z., Landreth G.E., Murakami S. (2012). FGF and ERK signaling coordinately regulate mineralization-related genes and play essential roles in osteocyte differentiation. J. Bone Min. Metab..

[32] Drissi H., Pouliot A., Koolloos C., Stein J.L., Lian J.B., Stein G.S., van Wijnen A.J. (2002). 1,25-(OH)2-vitamin D3 suppresses the bone-related *Runx2*/*Cbfa1* gene promoter. Exp. Cell Res..

[33] Meyer M.B., Benkusky N.A., Lee C.H., Pike J.W. (2014). Genomic determinants of gene regulation by 1,25-dihydroxyvitamin D3 during osteoblast-lineage cell differentiation. J. Boil. Chem..

[34] Underwood K.F., D’Souza D.R., Mochin-Peters M., Pierce A.D., Kommineni S., Choe M., Bennett J., Gnatt A., Habtemariam B., MacKerell A.D. (2012). Regulation of Runx2 transcription factor-DNA interactions and cell proliferation by vitamin D3 (cholecalciferol) prohormone activity. J. Bone Min. Res. Off. J. Am. Soc. Bone Min. Res..

[35] Fleet J.C., DeSmet M., Johnson R., Li Y. (2012). Vitamin D and cancer: A review of molecular mechanisms. Biochem. J.

[36] Welsh J. (2012). Cellular and molecular effects of vitamin D on carcinogenesis. Arch. Biochem. Biophys..

[37] Thompson L., Wang S., Tawfik O., Templeton K., Tancabelic J., Pinson D., Anderson H.C., Keighley J., Garimella R. (2012). Effect of 25-hydroxyvitamin D3 and 1α,25-dihydroxyvitamin D3 on differentiation and apoptosis of human osteosarcoma cell lines. J. Orthop. Res. Off. Publ. Orthop. Res. Soc..

[38] Garimella R., Eskew J., Bhamidi P., Vielhauer G., Hong Y., Clarke Anderson H., Tawfik O., Rowe P. (2013). Biological characterization of preclinical bioluminescent osteosarcoma orthotopic mouse (BOOM) model: A multi-modality approach. J. Bone Oncol..

[39] Wang S. (2010). Expression of Vitamin D Target Genes and Proteins in Human Osteosarcoma Cell Line, 143B in Response to 1α,25-Dihydroxyvitamin D3.

[40] Nakayama F., Muller K., Hagiwara A., Ridi R., Akashi M., Meineke V. (2008). Involvement of intracellular expression of FGF12 in radiation-induced apoptosis in mast cells. J. Radiat. Res..

[41] Wu X., Liu T., Fang O., Leach L.J., Hu X., Luo Z. (2014). miR-194 suppresses metastasis of non-small cell lung cancer through regulating expression of BMP1 and p27(kip1). Oncogene.

[42] Peehl D.M., Shinghal R., Nonn L., Seto E., Krishnan A.V., Brooks J.D., Feldman D. (2004). Molecular activity of 1,25-dihydroxyvitamin D3 in primary cultures of human prostatic epithelial cells revealed by cDNA microarray analysis. J. Steroid Biochem. Mol. Biol..

[43] Hoftijzer H.C., Liu Y.Y., Morreau H., van Wezel T., Pereira A.M., Corssmit E.P., Romijn J.A., Smit J.W. (2009). Retinoic acid receptor and retinoid X receptor subtype expression for the differential diagnosis of thyroid neoplasms. Eur. J. Endocrinol. Eur. Fed. Endocr. Soc..

[44] Lupov I.P., Voiles L., Han L., Schwartz A., De La Rosa M., Oza K., Pelloso D., Sahu R.P., Travers J.B., Robertson M.J. (2011). Acquired STAT4 deficiency as a consequence of cancer chemotherapy. Blood.

[45] Kahn R.A., Volpicelli-Daley L., Bowzard B., Shrivastava-Ranjan P., Li Y., Zhou C., Cunningham L. (2005). ARF family GTPases: Roles in membrane traffic and microtubule dynamics. Biochem. Soc. Trans..

[46] Jin H., Jin X., Cao B., Wang W. (2016). Berberine affects osteosarcoma via downregulating the caspase-1/IL-1β signaling axis. Oncol. Rep..

[47] Chaudhry P., Fabi F., Singh M., Parent S., Leblanc V., Asselin E. (2014). Prostate apoptosis response-4 mediates TGF-β-induced epithelial-to-mesenchymal transition. Cell Death Dis..

[48] Saladi S.V., Keenen B., Marathe H.G., Qi H., Chin K.V., de la Serna I.L. (2010). Modulation of extracellular matrix/adhesion molecule expression by BRG1 is associated with increased melanoma invasiveness. Mol. Cancer.

[49] Shi J., Whyte W.A., Zepeda-Mendoza C.J., Milazzo J.P., Shen C., Roe J.S., Minder J.L., Mercan F., Wang E., Eckersley-Maslin M.A. (2013). Role of SWI/SNF in acute leukemia maintenance and enhancer-mediated myc regulation. Genes Dev..

[50] Ondrusova L., Vachtenheim J., Reda J., Zakova P., Benkova K. (2013). MITF-Independent pro-survival role of BRG1-containing SWI/SNF complex in melanoma cells. PLoS ONE.

[51] Liu X., Tian X., Wang F., Ma Y., Kornmann M., Yang Y. (2014). BRG1 promotes chemoresistance of pancreatic cancer cells through crosstalking with AKT signalling. Eur. J. Cancer.

[52] Kothandapani A., Gopalakrishnan K., Kahali B., Reisman D., Patrick S.M. (2012). Downregulation of SWI/SNF chromatin remodeling factor subunits modulates cisplatin cytotoxicity. Exp. Cell Res..

[53] Seth-Vollenweider T., Joshi S., Dhawan P., Sif S., Christakos S. (2014). Novel mechanism of negative regulation of 1,25-dihydroxyvitamin D3-induced 25-hydroxyvitamin D3 24-hydroxylase (Cyp24a1) transcription: Epigenetic modification involving cross-talk between protein-arginine methyltransferase 5 and the SWI/SNF complex. J. Boil. Chem..

[54] Watanabe T., Semba S., Yokozaki H. (2011). Regulation of pten expression by the SWI/SNF chromatin-remodelling protein BRG1 in human colorectal carcinoma cells. Br. J. Cancer.

[55] Ohta A., Gorelik E., Prasad S.J., Ronchese F., Lukashev D., Wong M.K., Huang X., Caldwell S., Liu K., Smith P. (2006). A2A adenosine receptor protects tumors from antitumor T cells. Proc. Natl. Acad. Sci. USA.

[56] Hausler S.F., Montalban del Barrio I., Strohschein J., Anoop Chandran P., Engel J.B., Honig A., Ossadnik M., Horn E., Fischer B., Krockenberger M. (2011). Ectonucleotidases CD39 and CD73 on OVCA cells are potent adenosine-generating enzymes responsible for adenosine receptor 2A-dependent suppression of T cell function and NK cell cytotoxicity. Cancer Immunol. Immunother..

[57] Muller-Haegele S., Muller L., Whiteside T.L. (2014). Immunoregulatory activity of adenosine and its role in human cancer progression. Expert Rev. Clin. Immunol..

[58] Denburg M.R., Kalkwarf H.J., de Boer I.H., Hewison M., Shults J., Zemel B.S., Stokes D., Foerster D., Laskin B., Ramirez A. (2013). Vitamin D bioavailability and catabolism in pediatric chronic kidney disease. Pediatr. Nephrol..

[59] Kim H.J., Kim J.H., Bae S.C., Choi J.Y., Ryoo H.M. (2003). The protein kinase C pathway plays a central role in the fibroblast growth factor-stimulated expression and transactivation activity of Runx2. J. Boil. Chem..

[60] Cohen-Solal K.A., Boregowda R.K., Lasfar A. (2015). Runx2 and the PI3K/AKT axis reciprocal activation as a driving force for tumor progression. Mol. Cancer.

[61] Hollberg K., Marsell R., Norgard M., Larsson T., Jonsson K.B., Andersson G. (2008). Osteoclast polarization is not required for degradation of bone matrix in rachitic FGF23 transgenic mice. Bone.

[62] Babkina I.V., Osipov D.A., Solovyov Y.N., Bulycheva I.V., Machak G.N., Aliev M.D., Kushlinsky N.E. (2009). Endostatin, placental growth factor, and fibroblast growth factors-1 and -2 in the sera of patients with primary osteosarcomas. Bull. Exp. Boil. Med..

[63] Guagnano V., Kauffmann A., Wohrle S., Stamm C., Ito M., Barys L., Pornon A., Yao Y., Li F., Zhang Y. (2012). FGFR genetic alterations predict for sensitivity to NVP-BGJ398, a selective pan-FGFR inhibitor. Cancer Discov..

[64] Solan J.L., Deftos L.J., Goding J.W., Terkeltaub R.A. (1996). Expression of the nucleoside triphosphate pyrophosphohydrolase PC-1 is induced by basic fibroblast growth factor (BFGF) and modulated by activation of the protein kinase A and C pathways in osteoblast-like osteosarcoma cells. J. Bone Min. Res. Off. J. Am. Soc. Bone Min. Res..

[65] Liu S., Tang W., Fang J., Ren J., Li H., Xiao Z., Quarles L.D. (2009). Novel regulators of FGF23 expression and mineralization in Hyp bone. Mol. Endocrinol..

[66] Anderson H.C., Harmey D., Camacho N.P., Garimella R., Sipe J.B., Tague S., Bi X., Johnson K., Terkeltaub R., Millan J.L. (2005). Sustained osteomalacia of long bones despite major improvement in other hypophosphatasia-related mineral deficits in tissue nonspecific alkaline phosphatase/nucleotide pyrophosphatase phosphodiesterase 1 double-deficient mice. Am. J. Pathol..

[67] Johnson K., Pritzker K., Goding J., Terkeltaub R. (2001). The nucleoside triphosphate pyrophosphohydrolase isozyme PC-1 directly promotes cartilage calcification through chondrocyte apoptosis and increased calcium precipitation by mineralizing vesicles. J. Rheumatol..

[68] Garimella R., Washington L., Isaacson J., Vallejo J., Spence M., Tawfik O., Rowe P., Brotto M., Perez R. (2014). Extracellular membrane vesicles derived from 143B osteosarcoma cells contain pro-osteoclastogenic cargo: A novel communication mechanism in osteosarcoma bone microenvironment. Transl. Oncol..

[69] Vallet S., Anderson K.C. (2011). CCR1 as a target for multiple myeloma. Expert Opin. Ther. Targets.

[70] Pratap J., Javed A., Languino L.R., van Wijnen A.J., Stein J.L., Stein G.S., Lian J.B. (2005). The Runx2 osteogenic transcription factor regulates matrix metalloproteinase 9 in bone metastatic cancer cells and controls cell invasion. Mol. Cell. Biol..

[71] Pratap J., Imbalzano K.M., Underwood J.M., Cohet N., Gokul K., Akech J., van Wijnen A.J., Stein J.L., Imbalzano A.N., Nickerson J.A. (2009). Ectopic Runx2 expression in mammary epithelial cells disrupts formation of normal ACINI structure: Implications for breast cancer progression. Cancer Res..

[72] Riminucci M., Kuznetsov S.A., Cherman N., Corsi A., Bianco P., Gehron Robey P. (2003). Osteoclastogenesis in fibrous dysplasia of bone: In situ and in vitro analysis of IL-6 expression. Bone.

[73] Barnes G.L., Javed A., Waller S.M., Kamal M.H., Hebert K.E., Hassan M.Q., Bellahcene A., Van Wijnen A.J., Young M.F., Lian J.B. (2003). Osteoblast-related transcription factors Runx2 (Cbfa1/AML3) and MSX2 mediate the expression of bone sialoprotein in human metastatic breast cancer cells. Cancer Res..

[74] Akech J., Wixted J.J., Bedard K., van der Deen M., Hussain S., Guise T.A., van Wijnen A.J., Stein J.L., Languino L.R., Altieri D.C. (2010). Runx2 association with progression of prostate cancer in patients: Mechanisms mediating bone osteolysis and osteoblastic metastatic lesions. Oncogene.

[75] Wai P.Y., Mi Z., Gao C., Guo H., Marroquin C., Kuo P.C. (2006). ETS1 and Runx2 regulate transcription of a metastatic gene, osteopontin, in murine colorectal cancer cells. J. Boil. Chem..

[76] Hsu Y.L., Huang M.S., Yang C.J., Hung J.Y., Wu L.Y., Kuo P.L. (2011). Lung tumor-associated osteoblast-derived bone morphogenetic protein-2 increased epithelial-to-mesenchymal transition of cancer by Runx2/snail signaling pathway. J. Boil. Chem..

[77] Van der Deen M., Akech J., Lapointe D., Gupta S., Young D.W., Montecino M.A., Galindo M., Lian J.B., Stein J.L., Stein G.S. (2012). Genomic promoter occupancy of runt-related transcription factor Runx2 in osteosarcoma cells identifies genes involved in cell adhesion and motility. J. Boil. Chem..

[78] Bellido T., Ali A.A., Plotkin L.I., Fu Q., Gubrij I., Roberson P.K., Weinstein R.S., O’Brien C.A., Manolagas S.C., Jilka R.L. (2003). Proteasomal degradation of Runx2 shortens parathyroid hormone-induced anti-apoptotic signaling in osteoblasts. A putative explanation for why intermittent administration is needed for bone anabolism. J. Boil. Chem..

[79] Wan X., Kim S.Y., Guenther L.M., Mendoza A., Briggs J., Yeung C., Currier D., Zhang H., Mackall C., Li W.J. (2009). β4 integrin promotes osteosarcoma metastasis and interacts with ezrin. Oncogene.

[80] Hoshino A., Costa-Silva B., Shen T.L., Rodrigues G., Hashimoto A., Tesic Mark M., Molina H., Kohsaka S., Di Giannatale A., Ceder S. (2015). Tumour exosome integrins determine organotropic metastasis. Nature.

[81] Lim M., Zhong C., Yang S., Bell A.M., Cohen M.B., Roy-Burman P. (2010). Runx2 regulates survivin expression in prostate cancer cells. Lab. Investing. J. Tech. Methods Pathol..

[82] Davies J., Heeb H., Garimella R., Templeton K., Pinson D., Tawfik O. (2012). Vitamin D receptor, retinoid X receptor, KI-67, survivin, and ezrin expression in canine osteosarcoma. Vet. Med. Int..

[83] Shoeneman J.K., Ehrhart E.J., Eickhoff J.C., Charles J.B., Powers B.E., Thamm D.H. (2012). Expression and function of survivin in canine osteosarcoma. Cancer Res..

[84] Gupta A., Cao W., Chellaiah M.A. (2012). Integrin αvβ3 and CD44 pathways in metastatic prostate cancer cells support osteoclastogenesis via a Runx2/Smad5/receptor activator of NF-κB ligand signaling axis. Mol. Cancer.

[85] Liu W., Toyosawa S., Furuichi T., Kanatani N., Yoshida C., Liu Y., Himeno M., Narai S., Yamaguchi A., Komori T. (2001). Overexpression of Cbfa1 in osteoblasts inhibits osteoblast maturation and causes osteopenia with multiple fractures. J. Cell Boil..

[86] Martin J.W., Zielenska M., Stein G.S., van Wijnen A.J., Squire J.A. (2011). The role of Runx2 in osteosarcoma oncogenesis. Sarcoma.

[87] Irizarry R.A., Hobbs B., Collin F., Beazer-Barclay Y.D., Antonellis K.J., Scherf U., Speed T.P. (2003). Exploration, normalization, and summaries of high density oligonucleotide array probe level data. Biostatistics.

[88] Gunewardena S., Walesky C., Apte U. (2015). Global gene expression changes in liver following hepatocyte nuclear factor 4α deletion in adult mice. Genomics Data.

[89] Manzardo A.M., Gunewardena S., Wang K., Butler M.G. (2014). Exon microarray analysis of human dorsolateral prefrontal cortex in alcoholism. Alcohol. Clin. Exp. Res..

[90] Livak K.J., Schmittgen T.D. (2001). Analysis of relative gene expression data using real-time quantitative PCR and the 2^−ΔΔ*C*t^ method. Methods.

