# Supplementary material for: Vitamin D Impacts the Expression of Runx2 Target Genes and Modulates Inflammation, Oxidative Stress and Membrane Vesicle Biogenesis Gene Networks in 143B Osteosarcoma Cells
Source: Int J Mol Sci. 2017 Mar 16;18(3):642. doi: 10.3390/ijms18030642 (PMC5372654; doi:10.3390/ijms18030642)
Supplement: Supplementary file 1 [file ijms-18-00642-s001.zip › SF2.pptx]

## Slide 1
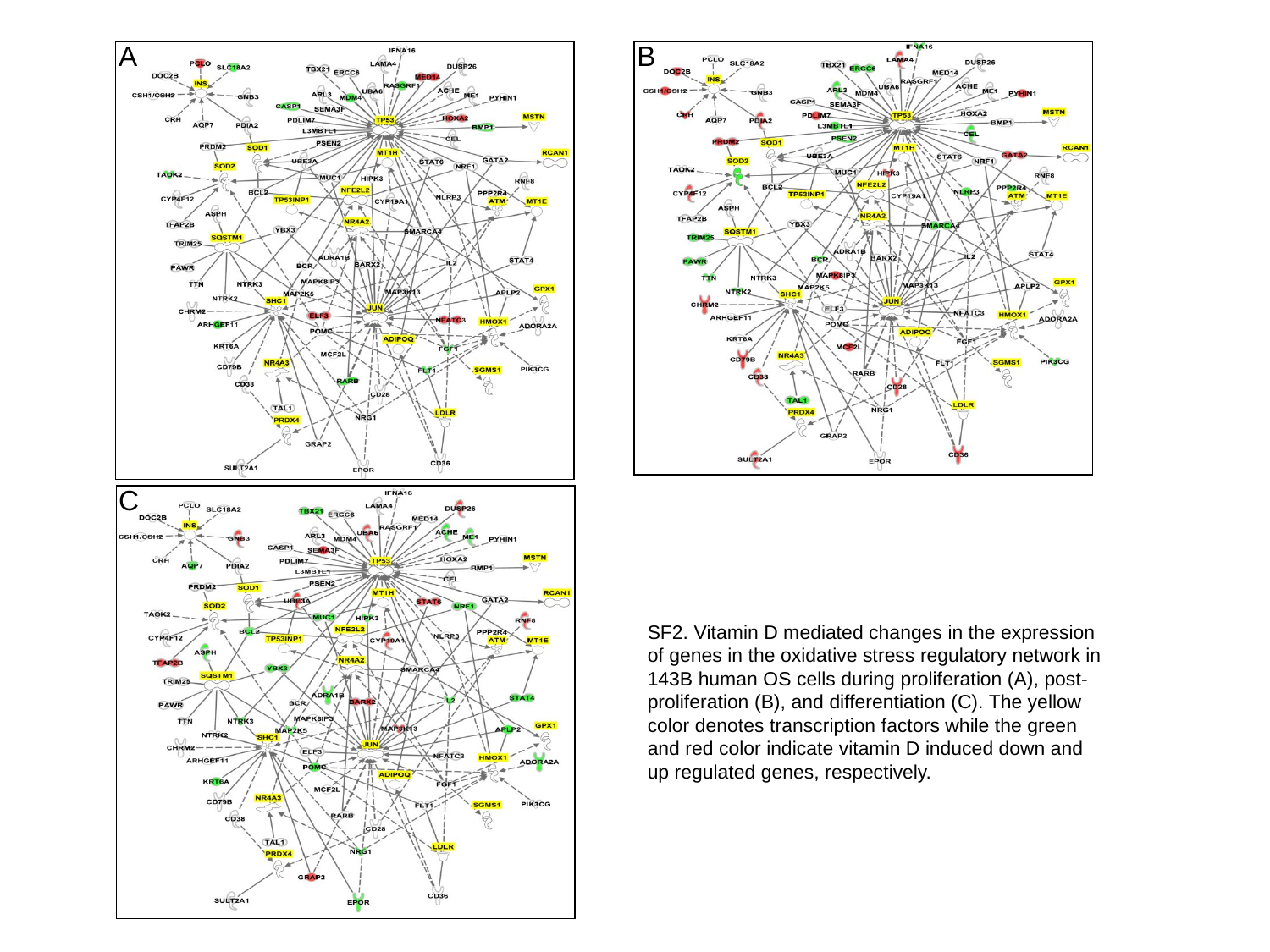

A
B
C
SF2. Vitamin D mediated changes in the expression of genes in the oxidative stress regulatory network in 143B human OS cells during proliferation (A), post-proliferation (B), and differentiation (C). The yellow color denotes transcription factors while the green and red color indicate vitamin D induced down and up regulated genes, respectively.
