# Supplementary material for: Vitamin D Impacts the Expression of Runx2 Target Genes and Modulates Inflammation, Oxidative Stress and Membrane Vesicle Biogenesis Gene Networks in 143B Osteosarcoma Cells
Source: Int J Mol Sci. 2017 Mar 16;18(3):642. doi: 10.3390/ijms18030642 (PMC5372654; doi:10.3390/ijms18030642)
Supplement: Supplementary file 1 [file ijms-18-00642-s001.zip › SF4.pptx]

## Slide 1
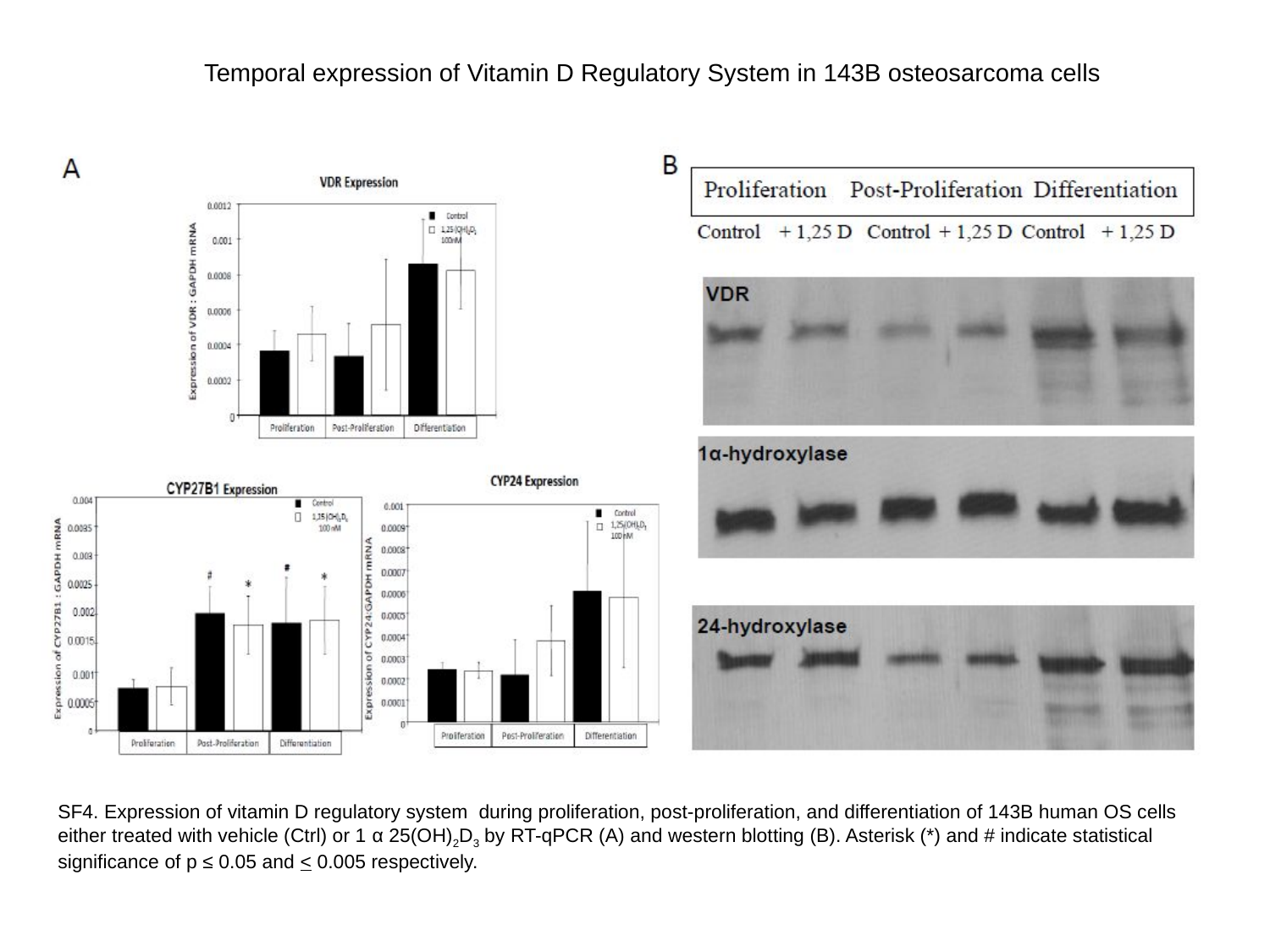

Temporal expression of Vitamin D Regulatory System in 143B osteosarcoma cells
#
SF4. Expression of vitamin D regulatory system during proliferation, post-proliferation, and differentiation of 143B human OS cells either treated with vehicle (Ctrl) or 1 α 25(OH)2D3 by RT-qPCR (A) and western blotting (B). Asterisk (*) and # indicate statistical significance of p ≤ 0.05 and < 0.005 respectively.
