# Supplementary material for: Vitamin D Impacts the Expression of Runx2 Target Genes and Modulates Inflammation, Oxidative Stress and Membrane Vesicle Biogenesis Gene Networks in 143B Osteosarcoma Cells
Source: Int J Mol Sci. 2017 Mar 16;18(3):642. doi: 10.3390/ijms18030642 (PMC5372654; doi:10.3390/ijms18030642)
Supplement: Supplementary file 1 [file ijms-18-00642-s001.zip › SF5.pptx]

## Slide 1
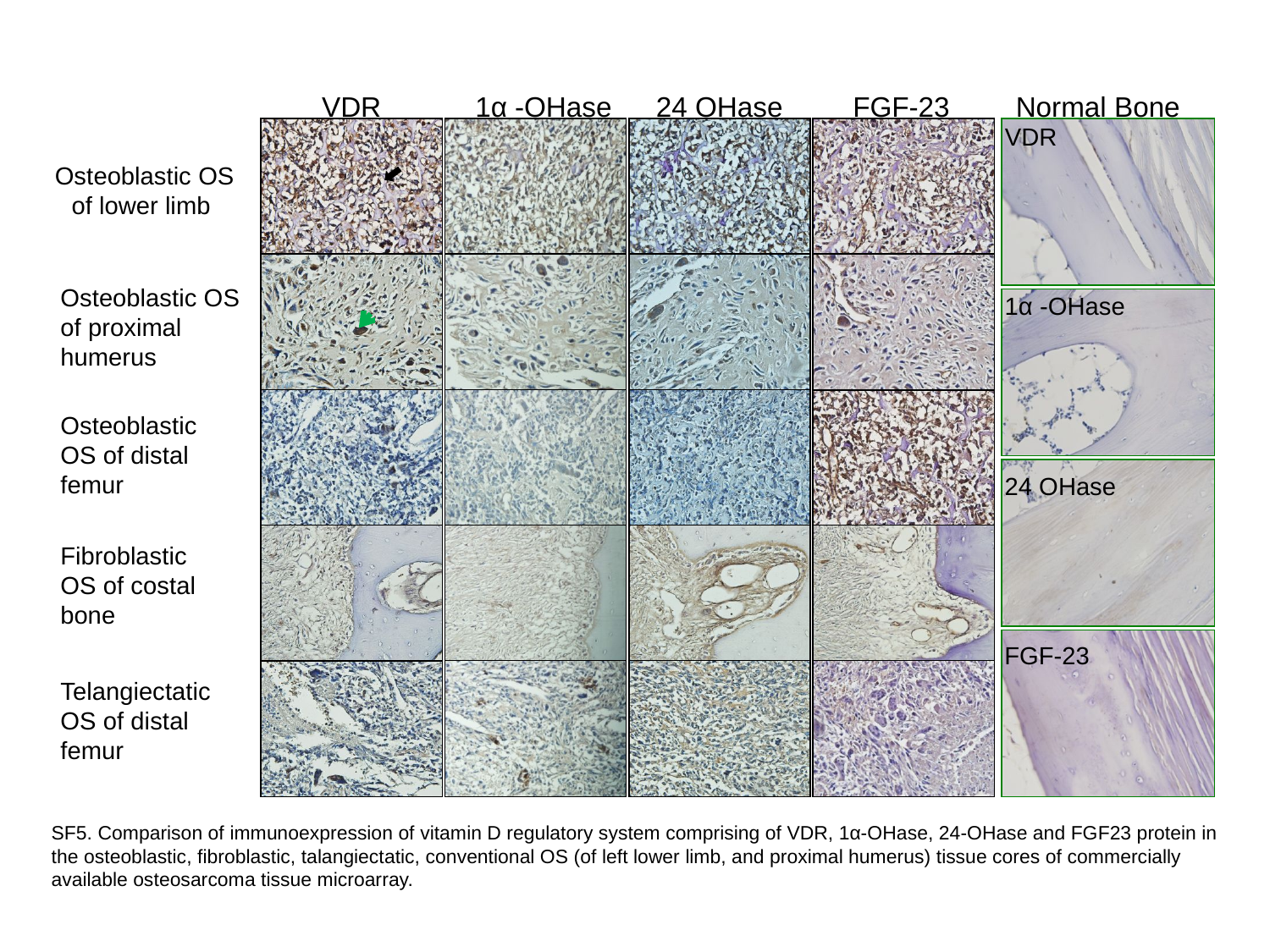

VDR
1α -OHase
24 OHase
FGF-23
Normal Bone
VDR
VDR
Osteoblastic OS of lower limb
Osteoblastic OS of proximal humerus
1α -OHase
Osteoblastic
OS of distal
femur
24 OHase
Fibroblastic
OS of costal
bone
FGF-23
Telangiectatic
OS of distal
femur
SF5. Comparison of immunoexpression of vitamin D regulatory system comprising of VDR, 1α-OHase, 24-OHase and FGF23 protein in the osteoblastic, fibroblastic, talangiectatic, conventional OS (of left lower limb, and proximal humerus) tissue cores of commercially available osteosarcoma tissue microarray.
