# Supplementary material for: Vitamin D Impacts the Expression of Runx2 Target Genes and Modulates Inflammation, Oxidative Stress and Membrane Vesicle Biogenesis Gene Networks in 143B Osteosarcoma Cells
Source: Int J Mol Sci. 2017 Mar 16;18(3):642. doi: 10.3390/ijms18030642 (PMC5372654; doi:10.3390/ijms18030642)
Supplement: Supplementary file 1 [file ijms-18-00642-s001.zip › SF6.pptx]

## Slide 1
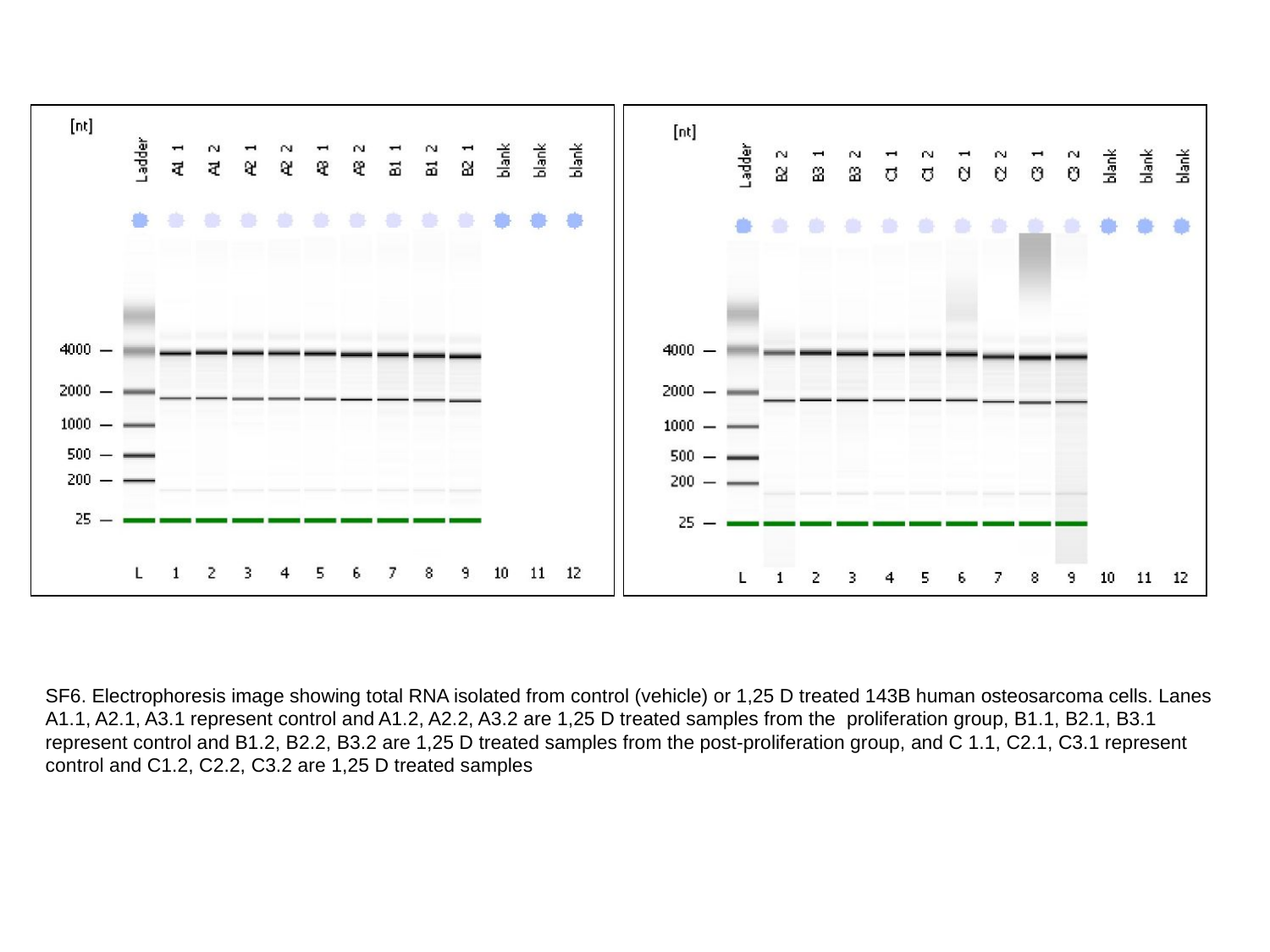

SF6. Electrophoresis image showing total RNA isolated from control (vehicle) or 1,25 D treated 143B human osteosarcoma cells. Lanes A1.1, A2.1, A3.1 represent control and A1.2, A2.2, A3.2 are 1,25 D treated samples from the proliferation group, B1.1, B2.1, B3.1 represent control and B1.2, B2.2, B3.2 are 1,25 D treated samples from the post-proliferation group, and C 1.1, C2.1, C3.1 represent control and C1.2, C2.2, C3.2 are 1,25 D treated samples
