# Supplementary material for: Vitamin D Impacts the Expression of Runx2 Target Genes and Modulates Inflammation, Oxidative Stress and Membrane Vesicle Biogenesis Gene Networks in 143B Osteosarcoma Cells
Source: Int J Mol Sci. 2017 Mar 16;18(3):642. doi: 10.3390/ijms18030642 (PMC5372654; doi:10.3390/ijms18030642)
Supplement: Supplementary file 1 [file ijms-18-00642-s001.zip › Supplementary Table 1A-F.pdf]

ST 1A. Most significant vitamin D down regulated genes in 143 B osteosarcoma cells during proliferation

Symbol	Entrez Gene Name	Affymetrix	Fold change	p-value	Top Diseases and Functions
FGF12	fibroblast growth factor 12	207501_s_at	-1.369	1.95E-02	Cellular Development, Proliferation, Hematological System Development and Function
CAPN3	calpain 3	214475_x_at	-1.331	9.75E-03	Cellular Development, Cellular Growth and Proliferation, Hematological System Development and Function
THBS3	thrombospondin 3	209561_at	-1.293	3.81E-02	Cellular Development, Embryonic Development, Nervous System Development and Function
*KLK7[a]	kallikrein-related peptidase 7	205778_at	-1.261	1.07E-03	Cardiovascular Disease, Cellular Assembly and Organization, Developmental Disorder
TACSTD2	tumor-associated calcium signal transducer 2	202285_s_at	-1.261	2.90E-02	Cell Death and Survival, Embryonic Development, Cell-To-Cell Signaling and Interaction
BCL2L10	BCL2-like 10 (apoptosis facilitator)	221320_at	-1.258	1.94E-02	Cardiovascular Disease, Cellular Assembly and Organization, Developmental Disorder
MEPE	matrix extracellular phosphoglycoprotein	221150_at	-1.255	4.01E-02	Cell-To-Cell Signaling and Interaction, Cell Signaling, Molecular Transport
*HIC1[a]	hypermethylated in cancer 1	208461_at	-1.251	1.39E-02	Cellular Development, Cellular Growth and Proliferation, Hematological System Development and Function
*RARB[a,b,c]	retinoic acid receptor, beta	208530_s_at	-1.242	1.01E-02	Cell Death and Survival, Embryonic Development, Cell-To-Cell Signaling and Interaction
CASP1	caspase 1, apoptosis-related cysteine peptidase	209970_x_at	-1.237	1.27E-02	Cell Death and Survival, Embryonic Development, Cell-To-Cell Signaling and Interaction

Superscripts a, b, and c represent genes/proteins as biomarkers biomarker for (a) diagnosis, (b) efficacy, and (c) prognosis

ST 1B. Most significant vitamin D up regulated genes in 143 B osteosarcoma cells during proliferation

Symbol	Entrez Gene Name	Affymetrix ID	Fold Change	P value	Top diseases and Functions
FBN1	fibrillin 1	202766_s_at	1.273	2.23E-02	Cell Death and Survival, Embryonic Development, Cell-To-Cell Signaling and Interaction
NFATC3	nuclear factor of activated T-cells, cytoplasmic, calcineurin-dependent 3	210555_s_at	1.248	1.53E-02	Cell Death and Survival, Embryonic Development, Cell-To-Cell Signaling and Interaction
ADAM21[b]	ADAM metallopeptidase domain 21	207665_at	1.237	2.62E-02	Cellular Development, Cellular Growth and Proliferation, Hematological System Development and Function
HSPB8	heat shock 22kDa protein 8	221667_s_at	1.232	2.13E-02	Cell Death and Survival, Embryonic Development, Cell-To-Cell Signaling and Interaction
CCNT2	cyclin T2	214638_s_at	1.231	4.78E-02	Cardiovascular Disease, Cellular Assembly and Organization, Developmental Disorder
PHF7	PHD finger protein 7	215622_x_at	1.222	4.49E-02	Cardiovascular Disease, Cellular Assembly and Organization, Developmental Disorder
TLL2	tolloid-like 2	215008_at	1.219	2.18E-02	Cellular Development, Cellular Growth and Proliferation, Hematological System Development and Function
RARRES1[a]	retinoic acid receptor responder (tazarotene induced) 1	206391_at	1.213	2.37E-02	Cellular Development, Cellular Growth and Proliferation, Hematological System Development and Function
BCL2L14	BCL2-like 14 (apoptosis facilitator)	221241_s_at	1.208	3.15E-02	Cell Death and Survival, Embryonic Development, Cell-To-Cell Signaling and Interaction
TLR6	toll-like receptor 6	207446_at	1.205	1.07E-02	Cell Death and Survival, Embryonic Development, Cell-To-Cell Signaling and Interaction

Superscripts a, b, and c represent genes/proteins as biomarkers biomarker for (a) diagnosis, (b) efficacy, and (c) prognosis

ST 1C. Most significant vitamin D down regulated genes in 143 B osteosarcoma cells during post-proliferation

Symbol	Entrez Gene Name	Affymetrix ID	Fold change	P value	Top diseases and Functions
ARL3	ADP-ribosylation factor-like 3	213433_at	-1.365	3.75E-02	Hereditary Disorder, Ophthalmic Disease, Gene Expression
PTPRN	protein tyrosine phosphatase, receptor type, N	204945_at	-1.363	4.56E-03	Cell Signaling, Molecular Transport, Nucleic Acid Metabolism
ANKRD6	ankyrin repeat domain 6	204672_s_at	-1.361	3.23E-02	Hereditary Disorder, Ophthalmic Disease, Gene Expression
SMARCA4	SWI/SNF related, matrix associated, actin dependent regulator of chromatin, subfamily a, member 4	214360_at	-1.302	1.22E-02	Cell Death and Survival, Cell Signaling, Molecular Transport
CTNNA2	catenin (cadherin-associated protein), alpha 2	205373_at	-1.285	2.14E-02	Cell Death and Survival, Cell Signaling, Molecular Transport
KLK3[d]	kallikrein-related peptidase 3	204583_x_at	-1.28	9.14E-03	Cell Death and Survival, Cell Signaling, Molecular Transport
MMP28	matrix metallopeptidase 28	219909_at	-1.265	4.37E-02	Cell Death and Survival, Cell Signaling, Molecular Transport
ANK1	ankyrin 1, erythrocytic	205389_s_at	-1.256	4.71E-02	Cell Death and Survival, Cell Signaling, Molecular Transport
ERCC6	excision repair cross-complementing rodent repair deficiency, complementation group 6	207347_at	-1.24	4.43E-02	Cell Signaling, Molecular Transport, Nucleic Acid Metabolism
PAWR[c]	PRKC, apoptosis, WT1, regulator	214237_x_at	-1.236	1.41E-02	Cell Signaling, Molecular Transport, Nucleic Acid Metabolism

Superscripts c and d represent genes/proteins as biomarkers biomarker for (c) prognosis and (d) disease progression

ST 1D. Most significant vitamin D up regulated genes in 143 B osteosarcoma cells during post-proliferation

Symbol	Entrez Gene Name	Affymetrix ID	Fold change	P value	Top diseases and Functions
CACNB1	calcium channel, voltage-dependent, beta 1 subunit	210967_x_at	1.346	0.0339	Hereditary Disorder, Ophthalmic Disease, Gene Expression
CRH[b]	corticotropin releasing hormone	205629_s_at	1.34	0.0436	Cell Death and Survival, Cell Signaling, Molecular Transport
GPR20	G protein-coupled receptor 20	214510_at	1.327	0.00457	Cellular Function and Maintenance, Cell-To-Cell Signaling and Interaction, Cell Signaling
ATF5	activating transcription factor 5	217389_s_at	1.32	0.003	Cell Signaling, Molecular Transport, Nucleic Acid Metabolism
ABCA4	ATP-binding cassette, sub-family A (ABC1), member 4	210082_at	1.318	0.00239	Dermatological Diseases and Conditions, Developmental Disorder, Organismal Injury and Abnormalities
ACAP1	ArfGAP with coiled-coil, ankyrin repeat and PH domains 1	205212_s_at	1.312	0.0154	Cell Death and Survival, Organ Morphology, Hematological System Development and Function
MAPK8IP3	mitogen-activated protein kinase 8 interacting protein 3	216137_s_at	1.278	0.0306	Cell Death and Survival, Organ Morphology, Hematological System Development and Function
SFRP5[a]	secreted frizzled-related protein 5	207468_s_at	1.275	0.0294	Hereditary Disorder, Ophthalmic Disease, Gene Expression
HOXB8	homeobox B8	221278_at	1.27	0.0386	Dermatological Diseases and Conditions, Developmental Disorder, Organismal Injury and Abnormalities
GPR162	G protein-coupled receptor 162	205056_s_at	1.264	0.00865	Cellular Function and Maintenance, Cell-To-Cell Signaling and Interaction, Cell Signaling

Superscripts a and b represent genes/proteins as biomarkers biomarker for (a) diagnosis, and (b) efficacy

ST 1E. Most significant vitamin D down regulated genes in 143 B osteosarcoma cells during differentiation

Symbol	Entrez Gene Name	Affymetrix ID	Fold Change	p-value	Top Diseases and Function
ENTPD2	ectonucleoside triphosphate diphosphohydrolase 2	207372_s_at	-1.36	1.80E-02	Connective Tissue Disorders, Inflammatory Disease, Skeletal and Muscular Disorders
CASQ2	calsequestrin 2 (cardiac muscle)	207317_s_at	-1.346	1.34E-02	Immunological Disease, Organismal Injury and Abnormalities, Molecular Transport
NCR3	natural cytotoxicity triggering receptor 3	211010_s_at	-1.345	2.27E-03	Connective Tissue Disorders, Inflammatory Disease, Skeletal and Muscular Disorders
ABCC9	ATP-binding cassette, sub-family C (CFTR/MRP), member 9	208562_s_at	-1.316	1.08E-02	Immunological Disease, Organismal Injury and Abnormalities, Molecular Transport
STAT4	signal transducer and activator of transcription 4	206118_at	-1.311	1.04E-02	Connective Tissue Disorders, Inflammatory Disease, Skeletal and Muscular Disorders
FOXH1	forkhead box H1	207644_at	-1.285	3.71E-02	Nutritional Disease, Cellular Assembly and Organization, Cellular Movement
ARL4D	ADP-ribosylation factor-like 4D	203587_at	-1.281	8.31E-03	Connective Tissue Disorders, Developmental Disorder, Hereditary Disorder
ZNF225	zinc finger protein 225	207125_at	-1.28	3.04E-02	Cellular Development, Cell Morphology, Cellular Function and Maintenance
ZNF780A	zinc finger protein 780A	215570_s_at	-1.278	4.05E-02	Transcription regulation
ADORA2A	adenosine A2a receptor	205013_s_at	-1.269	1.22E-02	Immunological Disease, Organismal Injury and Abnormalities, Molecular Transport

ST 1F. Most significant vitamin D up regulated genes in 143 B osteosarcoma cells during differentiation

Symbol	Entrez Gene Name	Affymetrix ID	FC	P value	Top Diseases and Function
UBE3A	ubiquitin protein ligase E3A	214980_at	1.413	0.0104	Connective Tissue Disorders, Inflammatory Disease, Skeletal and Muscular Disorders
TFAP2B	transcription factor AP-2 beta (activating enhancer binding protein 2 beta)	214451_at	1.269	0.00766	Connective Tissue Disorders, Developmental Disorder, Hereditary Disorder
AJAP1	adherens junctions associated protein 1	215789_s_at	1.268	0.0412	Embryonic Development, Organ Development, Organismal Development
CDK19	cyclin-dependent kinase 19	211706_s_at	1.26	0.0183	Connective Tissue Disorders, Developmental Disorder, Hereditary Disorder
GTPBP1	GTP binding protein 1	205276_s_at	1.255	0.0288	Nutritional Disease, Cellular Assembly and Organization, Cellular Movement
PKLR	pyruvate kinase, liver and RBC	222078_at	1.249	0.0145	Connective Tissue Disorders, Inflammatory Disease, Skeletal and Muscular Disorders
RNF8	ring finger protein 8, E3 ubiquitin protein ligase	203161_s_at	1.248	0.0484	Connective Tissue Disorders, Developmental Disorder, Hereditary Disorder
STAT6[e]	signal transducer and activator of transcription 6, interleukin-4 induced	201332_s_at	1.246	0.0259	Connective Tissue Disorders, Inflammatory Disease, Skeletal and Muscular Disorders
CDH17	cadherin 17, LI cadherin (liver-intestine)	209847_at	1.241	0.0337	Nutritional Disease, Cellular Assembly and Organization, Cellular Movement
MAP3K13	mitogen-activated protein kinase kinase kinase 13	211083_s_at	1.241	0.00181	Immunological Disease, Organismal Injury and Abnormalities, Molecular Transport
KANK2	KN motif and ankyrin repeat domains 2	221068_at	1.227	0.0185	Nutritional Disease, Cellular Assembly and Organization, Cellular Movement

Superscript e represents genes/proteins as biomarkers biomarker for response to therapy
