# Supplementary material for: Vitamin D Impacts the Expression of Runx2 Target Genes and Modulates Inflammation, Oxidative Stress and Membrane Vesicle Biogenesis Gene Networks in 143B Osteosarcoma Cells
Source: Int J Mol Sci. 2017 Mar 16;18(3):642. doi: 10.3390/ijms18030642 (PMC5372654; doi:10.3390/ijms18030642)
Supplement: Supplementary file 1 [file ijms-18-00642-s001.zip › Supplementary Table 2.pptx]

## Slide 1
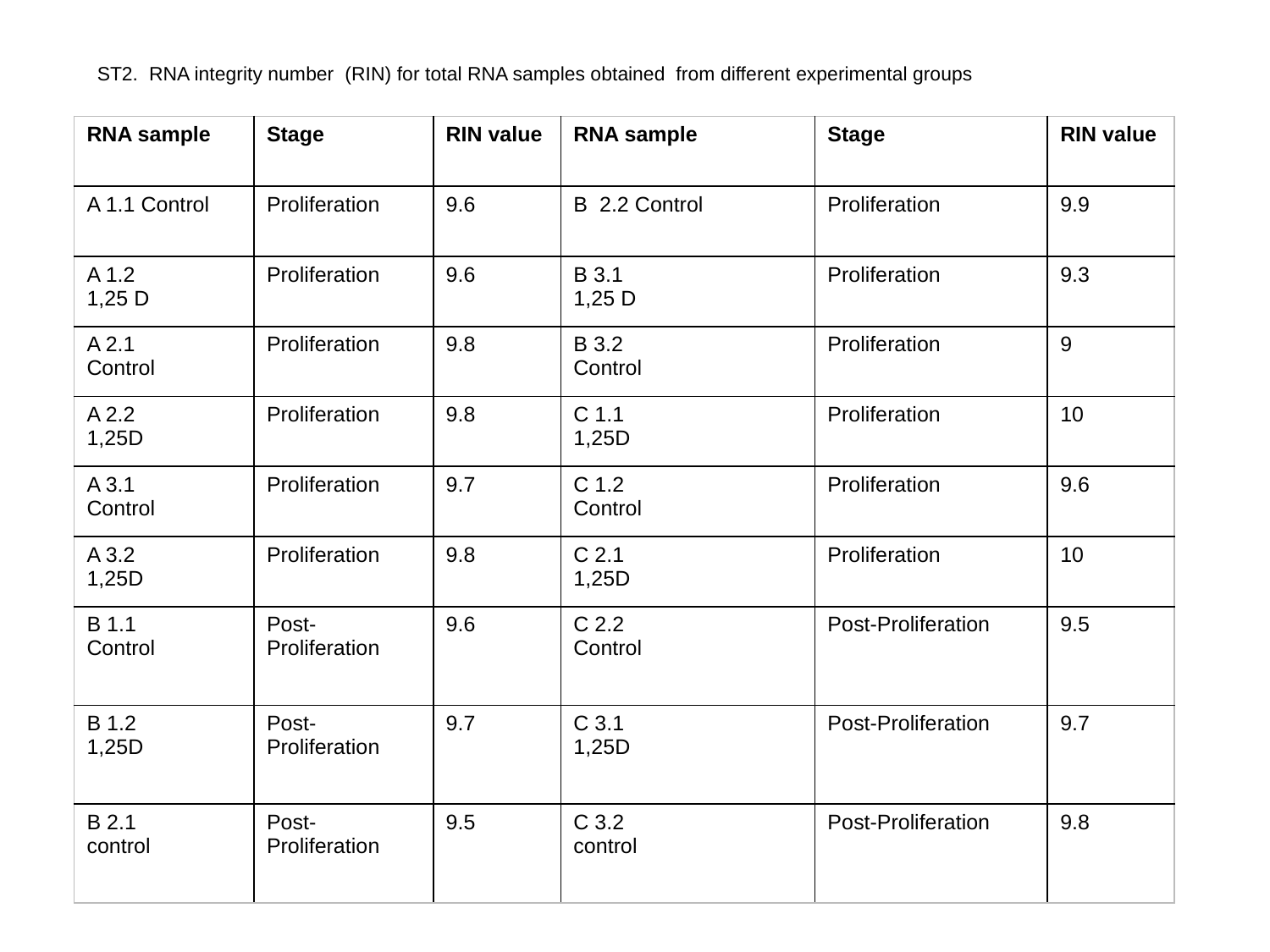

ST2. RNA integrity number (RIN) for total RNA samples obtained from different experimental groups
| RNA sample | Stage | RIN value | RNA sample | Stage | RIN value |
| --- | --- | --- | --- | --- | --- |
| A 1.1 Control | Proliferation | 9.6 | B 2.2 Control | Proliferation | 9.9 |
| A 1.2 1,25 D | Proliferation | 9.6 | B 3.1 1,25 D | Proliferation | 9.3 |
| A 2.1 Control | Proliferation | 9.8 | B 3.2 Control | Proliferation | 9 |
| A 2.2 1,25D | Proliferation | 9.8 | C 1.1 1,25D | Proliferation | 10 |
| A 3.1 Control | Proliferation | 9.7 | C 1.2 Control | Proliferation | 9.6 |
| A 3.2 1,25D | Proliferation | 9.8 | C 2.1 1,25D | Proliferation | 10 |
| B 1.1 Control | Post-Proliferation | 9.6 | C 2.2 Control | Post-Proliferation | 9.5 |
| B 1.2 1,25D | Post-Proliferation | 9.7 | C 3.1 1,25D | Post-Proliferation | 9.7 |
| B 2.1 control | Post-Proliferation | 9.5 | C 3.2 control | Post-Proliferation | 9.8 |
